# Heavy metal resistance in the Yanomami and Tunapuco microbiome

**DOI:** 10.1590/0074-02760230086

**Published:** 2023-11-10

**Authors:** Liliane Costa Conteville, Joseli Oliveira-Ferreira, Ana Carolina P Vicente

**Affiliations:** 1 Fundação Oswaldo Cruz-Fiocruz, Instituto Oswaldo Cruz, Laboratório de Genética Molecular de Microrganismos, Rio de Janeiro, RJ, Brasil; 2 Embrapa Pecuária Sudeste, São Carlos, SP, Brasil; 3 Fundação Oswaldo Cruz-Fiocruz, Instituto Oswaldo Cruz, Laboratório de Imunoparasitologia, Rio de Janeiro, RJ, Brasil

**Keywords:** gut microbiome, mercury resistance, silver resistance, hunter-gatherers, shotgun metagenomic sequencing

## Abstract

**BACKGROUND:**

The Amazon Region hosts invaluable and unique biodiversity as well as mineral resources. Consequently, large illegal and artisanal gold mining areas exist in indigenous territories. Mercury has been used in gold mining, and some has been released into the environment and atmosphere, primarily affecting indigenous people such as the Yanomami. In addition, other heavy metals have been associated with gold mining and other metal-dispersing activities in the region.

**OBJECTIVE:**

Investigate the gut microbiome of two semi-isolated groups from the Amazon, focusing on metal resistance.

**METHODS:**

Metagenomic data from the Yanomami and Tunapuco gut microbiome were assembled into contigs, and their putative proteins were searched against a database of metal resistance proteins.

**FINDINGS:**

Proteins associated with mercury resistance were exclusive in the Yanomami, while proteins associated with silver resistance were exclusive in the Tunapuco. Both groups share 77 non-redundant metal resistance (MR) proteins, mostly associated with multi-MR and operons with potential resistance to arsenic, nickel, zinc, copper, copper/silver, and cobalt/nickel. Although both groups harbour operons related to copper resistance, only the Tunapuco group had the *pco* operon.

**CONCLUSION:**

The Yanomami and Tunapuco gut microbiome shows that these people have been exposed directly or indirectly to distinct scenarios concerning heavy metals.

Heavy metals are naturally occurring elements found in the environment.^1^ And in biological systems, they play an essential role in carrying out various physiological and biochemical functions.^2^ ^,^ ^3^ However, the distribution and amount of heavy metals have increased significantly worldwide due to various anthropogenic activities such as mining, industrial practices, and the addition of metals to fertilisers and livestock feed.^4^ ^,^ ^5^ Thus, high concentrations of heavy metals have been detected in soils, sediments, and water bodies. The resulting selective pressure often leads to dramatic changes within their microbial populations and ecological functions.^6^ ^-^ ^11^ This process has exceeded the tolerable baseline level of natural heavy metal cycling in many ecosystems and is currently a problem not only for the environment but also for public health.^2^

Exposure of humans and animals to heavy metals can occur through water, air, food, or skin, and bioaccumulation of heavy metals can lead to various toxic effects on different tissues and organs.^12^ ^,^ ^13^ ^,^ ^14^ For example, arsenic, cadmium, and chromium are carcinogenic metals that can impair DNA synthesis and repair.^15^ ^,^ ^16^ To cope with high metal concentrations, microbes have evolved adaptation and resistance mechanisms such as metal resistance (MR) genes.^17^ These mechanisms are thought to result from billions of years of selection by geological heavy metals and later by anthropogenic influences.^2^ ^,^ ^18^

The Amazon hosts an invaluable and unique biodiversity of flora and fauna on Earth and is home to 173 ethnic groups, with indigenous territories occupying a significant portion of the region (27% of the forest area).^19^ Nevertheless, the Brazilian Amazon is home to the third largest area of artisanal mining in indigenous territories,^20^ where mercury (Hg) is used to combine with gold in the amalgamation process.^21^ Mercury is considered one of the ten most hazardous substances to health by the World Health Organization (WHO) due to its high toxicity^22^ and in the amalgamation process, some mercury is dispersed into aquatic environments, soils, and the atmosphere,^23^ ^,^ ^24^ affecting indigenous areas such as the Yanomami.^23^

The Yanomami are the largest indigenous, semi-isolated group in the Amazon that still maintains traditional subsistence practices (hunting, fishing, gathering, and swidden horticulture). Their land encompasses the Venezuela-Brazil border and spans two Brazilian states (Roraima and Amazonas).^24^ Reports indicate that this group has been exposed to mercury from gold mining since 1980,^25^ and it is a current and growing threat.^26^ The Yanomami are even more vulnerable because of their dependence on resources in this potentially contaminated environment. Fish in the Amazon, the main source of protein for traditional groups, is also contaminated with mercury,^27^ ^,^ ^28^ and dietary exposure is the major pathway for the accumulation of metals in the body. In addition to mercury, other heavy metals such as copper (Cu), zinc (Zn), arsenic (As), cadmium (Cd), and lead (Pb) have also been associated with gold mining.^29^ However, other metal-dispersing activities may also be occurring in the region. As our group has previously noted, metal contamination could result from the continuous discharge of batteries by the Yanomami on their land for decades.^30^

Tunapuco is a rural agriculturalist group situated in the central Andes, at an elevation between 2,500 and 3,100 m above sea level. Their diet is based on local agricultural products (mainly potatoes, oca, and mashua) and home-raised small animals.^31^ The Peruvian Andes hosts a wealth of mineral resources such as copper, silver, zinc, and gold, with silver mining being one of the region’s most important activities.^32^

In addition to the toxic effects caused by metal contamination, the gut microbiota of individuals may reflect their long-term metal exposure.^33^ However, studies of this nature are extremely limited, and we found none that focused on isolated or semi-isolated groups. Thus, to gain insight into this issue, we compared the gut microbiomes of two different groups, the Brazilian Yanomami and the Tunapuco, focusing on the differences in their heavy metal resistance profiles.

## MATERIALS AND METHODS

*Data used* - For this study, we analysed shotgun metagenomic data from the gut microbiome of the Yanomami (n = 15), a semi-isolated hunter-gatherer community from the Brazilian Amazon,^30^ and the Tunapuco (n = 12), a traditional agricultural community from the Andean highlands.^31^ The metagenomic data from both groups comprises paired-end sequences generated on Illumina sequencers and can be found in the National Centre for Biotechnology Information Sequence Read Archive (https://www.ncbi.nlm.nih.gov/sra) under the BioProjects PRJNA527208 and PRJNA268964.

*Metagenomic assemble and open reading frames (*ORFs*) prediction* - Each metagenome was independently subjected to *de novo* assembly through metaSPAdes v.3.13^34^ using default parameters and *k-mers* of size 21, 33 and 55. This step assembled the short reads of the metagenomes into 2,012,469 contigs (longer sequences). ORFs were predicted in these contigs with FragGeneScan v.1.31^35^ using the parameters “- w 1 -t complete”.

*Sequences classification* - The translated ORFs were matched to specific proteins using the Abricate program (https://github.com/tseemann/abricate). This program uses BLAST+^36^ to compare sequences with reference databases, and in this study, we used the BacMet2 database^17^ to analyse metal resistance proteins. The results from each search were filtered, so only proteins with more than 80% coverage and 80% identity were considered for further analysis.

To relate the identified proteins to the bacterial species of the Yanomami/Brazil microbiome, the contigs that showed similarities to MR proteins were taxonomically classified using the Kraken2 program.^37^

*Gene abundance and reads per kilobase per million sequenced reads (RPKM) calculation* - Metagenomic reads were aligned to the MR coding genes using BWA mem v0.7.17^38^ with default parameters. SAMtools v1.13^39^ was used for file manipulation and assessment of the reads abundance and length of each gene. Abundances were then normalised using a RPKM approach that normalises to the total number of reads sequenced per sample, not the total number of reads mapped.^40^ The following formula was used: RPKM = number of reads mapped to the ORF / (length of the ORF / 1000* total number of reads sequenced per sample / 1,000,000).

*Metals determinants operons* - To identify the presence of metal resistance operons, we surveyed the presence of coding genes in the metagenomes. Mercury resistance operon was determined by the *mer* operon, which consists of genes responsible for mercuric reduction (*merA*), periplasmic binding (*merP*), transport (*merTCEG*), and transcriptional regulation (*merRD*),^41^ but also genes associated with organomercury compounds degradation (*merB* and *merG*).^42^ Arsenic resistance operon was determined by the *ars* operon (inorganic arsenic), which has many variants but, at the minimum, it consists of genes responsible for arsenite efflux transporter ATPase subunit (*arsB*), arsenate reductase (*arsC*) and a metalloregulatory transcriptional repressor (*arsR*).^43^ The copper resistance operon was determined by a system for copper efflux (*cue*), a system for copper sensing (*cus*)^44^ and the *pco* operon.^45^ The cue system consists of the periplasmic multicopper oxidase (*cueO)*, the membrane-embedded ATP-driven copper efflux pumps (*copAB*), and the transcriptional regulator (*cueR*).^44^ ^,^ ^46^ The *cus* system has the potential to also confer silver resistance, and it includes a complex of proton gradient-driven efflux pump (*cusABC*) and regulators (*cusRS*).^44^ The *pco* operon consists of the multicopper oxidase (*pcoA*), the copper-binding protein (*pcoB*), metallochaperones (*pcoCE*), membrane components (*pcoD* and *pcoS*), and transcriptional regulator (*pcoR*).^45^ The zinc resistance operon was determined by the presence of proteins that control zinc homeostasis by importing and exporting Zn ions via Zn uptake systems.^47^ The proteins *znuABC* are ABC transporters that facilitate zinc ions uptake, which can be regulated by *zitB*, *zur*, or *soxS*.^47^ ^,^ ^48^ Chromate resistance operon was determined by the chromate ion transport (*chr*) superfamily, which consists of genes responsible for reduction (*nfsA* and *chrR/yieF*), transport (*chrA*), regulation (*chrB* and *chrF*) and superoxide dismutase (*chrC*).^49^ Nickel resistance operon was determined by the *nik* system of ABC transporters, which is involved in specific nickel uptake and is composed of a periplasmic binding protein (*nikA*), integral membrane components (*nikBC*), two ATPases (*nikDE*), and a repressor that avoids nickel overload.^50^ ^,^ ^51^ Silver resistance was determined by the presence of the *sil* operon, which consists of efflux pumps (*silCBA* and *silP*), binding proteins (*silF* and *silE*) and a two-component regulatory system (genes *silRS*).^52^ Cobalt and nickel resistance operon was determined by the *rcn* system, which is composed of a regulator (*rcnR*) that controls the transcription of cobalt and nickel exporters (*rcnAB*).^53^

## RESULTS

**Fig. 1:**
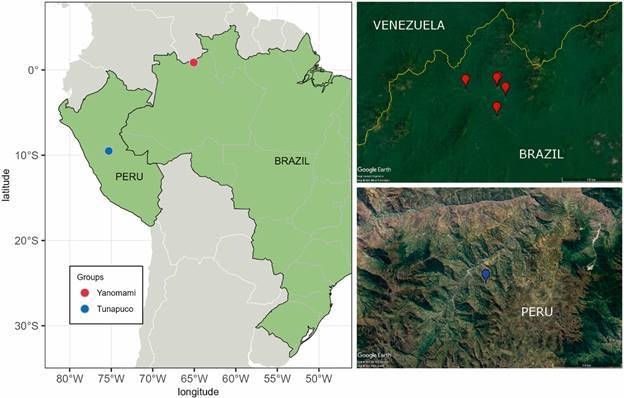
geographic locations and satellite images of the Yanomami and Tunapuco regions. Map generated in R and satellite images obtained from Google Earth.

We reanalysed the metagenomic data from the gut microbiome of Yanomami and Tunapuco individuals (Fig. 1). Although both are semi-isolated groups living within the Amazon Region, their lifeways and environments are startlingly different.

**Fig. 2:**
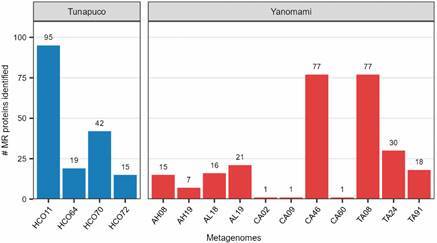
amount of metal resistance (MR) proteins identified per metagenome in the Tunapuco and Yanomami groups.

Putative proteins from the metagenomic contigs were screened for similarity with MR proteins from the BacMet2 database. Four of the 12 Tunapuco metagenomes and 11 of the 15 Yanomami metagenomes presented MR proteins (Fig. 2).

MR proteins with more copies in the Yanomami are encoded by *merT* and *merP* (identified eleven and nine times, respectively), followed by *pstB* and *chrA1* (identified eight times). *merT* was identified in four metagenomes, having up to five copies each, while *merP* was identified in five metagenomes with up to three copies each. *pstB* was identified in six metagenomes, duplicated in two of them. *chrA1* was duplicated in the four metagenomes in which it was identified. MR proteins with more copies in the Tunapuco are also the most prevalent, encoded by *arsR*, *cutA*, *yhcN,* and *zur*, each of which has been identified four times, once in each of the four metagenomes with MR proteins. These four proteins were also identified in the Yanomami.

**Fig. 3:**
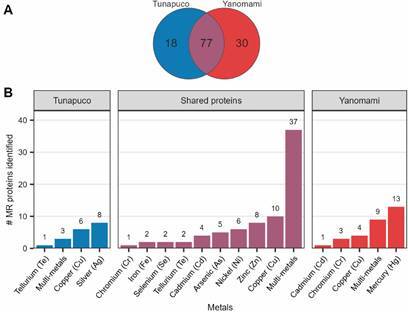
(A) Venn diagram and the (B) amount grouped by metals of the unique and shared metal resistance (MR) proteins between the Tunapuco and Yanomami groups.

Out of the 107 and 95 non-redundant MR proteins identified in the Yanomami and Tunapuco, respectively, 77 are shared between the two groups (Fig. 3A), with most being associated with resistance to multi-metals (two or more metals) (Fig. 3B). The majority of unique MR proteins in the Yanomami are associated with mercury resistance, while the majority of unique MR proteins in the Tunapuco are associated with silver resistance. It is noteworthy that the Tunapuco did not present any protein capable of conferring resistance to mercury, and the Yanomami did not present any protein capable of conferring resistance to silver (Fig. 3B).

Contigs that potentially encode MR proteins in the Yanomami belong to the phylum *Proteobacteria* (170 contigs) and *Firmicutes* (four contigs), while in the Tunapuco belong exclusively to *Proteobacteria* (102 contigs). Taxonomic information regarding the contigs from both groups can be found in Supplementary data (Tables I-II). In the Yanomami, the most abundant genus from the *Proteobacteria* phylum was *Escherichia* (114 contigs with 80 MR proteins), followed by *Ralstonia* (18 contigs with seven MR genes). From the phylum *Firmicutes*, the genera identified were *Enterococcus* (three contigs with four MR genes) and *Streptococcus* (one contig with one MR gene). In the Tunapuco, the contigs were classified as belonging to the genera *Escherichia* (81 contigs with 91 MR proteins), and the family *Enterobacteriaceae* (21 contigs with 22 MR proteins).

**Fig. 4:**
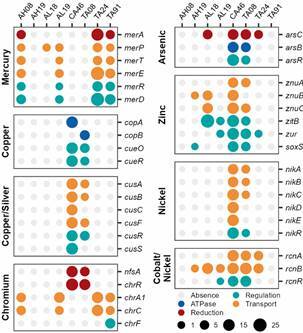
dot chart representing the coding genes of the operons identified in each Yanomami metagenome coloured by function and grouped by the metal(s) in which they have the potential to confer resistance. The size of each dot represents the reads per kilobase per million sequenced reads (RPKM) abundance of each gene in each metagenome.

**Fig. 5:**
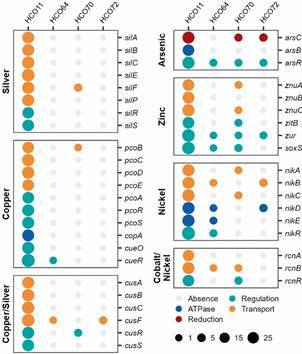
dot chart representing the coding genes of the operons identified in each Tunapuco metagenome coloured by function and grouped by the metal(s) in which they have the potential to confer resistance. The size of each dot represents the reads per kilobase per million sequenced reads (RPKM) abundance of each gene in each metagenome.

We further investigated the presence and abundance of heavy metals operons in the metagenomes [Figs 4, 5, Supplementary data (Table III)]. We identified nine operons or systems (*mer*, *ars*, *chr*, *nik*, *znu*, *cue/cop/pco* and *sil*) that have the potential to confer resistance to mercury, arsenic, chromium, nickel, zinc, copper and silver, respectively. We also identified two operons (*cus* and *rcn*) associated with multiple metals (copper/silver and cobalt/nickel).

The *mer* operon was the most predominant in the Yanomami, being identified in the metagenomes AH08, AL19, TA24, and TA91, which have putative proteins responsible for the main functions necessary for mercury resistance, but the mechanisms for organomercury compound degradation (*merB* and *merG*) were not identified (Fig. 4). The metagenome AL19 does not have the *merA* protein for mercuric reduction and also has the lowest RPKM values for the other proteins from the *mer* operon. All contigs harbouring putative proteins from the *mer* operon were classified as *Proteobacteria*, mainly *Aeromonas*, *Burkholderia*, and *Paraburkholderia* [Supplementary data (Table I)]. These four metagenomes also harbour putative proteins involved in chromium transport (*chrA1* and *chrC*) in contigs classified as *Ralstonia*. In contrast, the metagenomes CA46 and TA08 harbour genes that encode proteins involved in chromium reduction (*nfsA* and *chrR*) in contigs that were classified as *Escherichia*, *Ralstonia*, *Paraburkholderia*, *Kinneretia*, and *Enterobacteriaceae* [Fig. 4, Supplementary data (Table I)].

Moreover, the Yanomami metagenomes CA46 and TA08 stand out for having the highest number of operons detected (nickel, cobalt/nickel, arsenic, zinc, copper, and copper/silver). Most contigs (64/70) harbouring these operons were classified as *Escherichia coli*. The same contig from metagenome CA46 harbours the complete *nik* and *ars* operons, while the genes from the *rcn* and *cus* operons were identified in different but also single contigs [Supplementary data (Table I)].

The unique proteins associated with silver resistance in the Tunapuco belonged to the *sil* operon. This operon and all the other operons identified in the Tunapuco group were complete in the metagenome HCO11 (Fig. 5). Compared with the other metagenomes, the putative proteins from these operons in the HCO11 had the highest RPKM values. Yanomami and Tunapuco harbour operons associated with copper resistance, but the *pco* operon was exclusively identified in the Tunapuco group. In the HCO11 metagenome, the complete *pco* and *nik* operons were identified in the same contig, while the complete *cus*, *ars* and *rcn* operons were identified each in a single contig (Fig. 5). These four contigs were classified as *E. coli* [Supplementary data (Table II)]*.* The other Tunapuco metagenomes also harbour proteins from these operons; however, they did not present the complete set. The metagenome HCO70 harbours partial operons associated with arsenic, zinc and cobalt/nickel resistance, the metagenome HCO72 harbours a partial operon associated with arsenic, and the metagenome HCO64 harbours a partial operon associated with nickel (Fig. 5).

## DISCUSSION

In general, the structure, composition, and diversity of gut microbiomes reflect the living conditions and environment of the individuals.^54^ Although heavy metals are naturally occurring elements found mainly in soil, heavy metal pollution in mining areas is a serious environmental and human health concern. In our study, we characterised heavy metal resistance genes/proteins associated with the gut microbiome of Yanomami and Tunapuco individuals.

The Yanomami is a semi-nomadic hunter-gatherer group from the Amazon. Although they explore a rich and highly biodiverse territory, gold mining has been practiced in their land for decades.^25^ This practice usually relies on a process that results in the dispersal of mercury into the atmosphere and environment.^55^ ^,^ ^56^ Remarkably, we identified the *mer* operon (mercury resistance) and other heavy metal-associated operons in the Yanomami microbiome. The Tunapuco is an agricultural group that inhabits a large area in the Central Andes (Panao, Pachitea, province of Huánuco) where mining activities have not been reported.^31^ ^,^ ^57^ Although most Tunapuco metagenomes did not show any MR protein, we identified complete heavy-metal-associated operons in the microbiome of one individual and partial operons in the microbiomes of three other individuals. These operons include the *sil* and *pco* operons (silver and copper resistance), not harboured by the Yanomami. Notably, Peru has been one of the world’s biggest producers of silver, copper, and zinc.^32^ Identifying MR proteins related to heavy metals associated with mining practices could suggest that the Yanomami and Tunapuco groups have been exposed to diverse heavy metals, which may have cumulative effects and cause higher toxicities.^13^

In the Yanomami, most identified proteins have been associated with resistance to toxic metals such as mercury, copper, and zinc, while the most identified proteins in the Tunapuco have been associated with copper, zinc, and nickel.

The mer operon, only identified in the Yanomami, included the genes (*merT* and *merP*) required for the full expression of mercury resistance,^58^ which had the largest number of copies in the Yanomami. The narrow-spectrum mercury resistance *merRTPADE* operon confers resistance to inorganic mercury, while the broad-spectrum mercury resistance *merRTPAGBDE* operon confers resistance to inorganic and organic mercury. Both include the central enzyme in the microbial mercury detoxification system: the mercuric reductase (MERA) protein, which catalyses the reduction of Hg(II) to volatile Hg(0). Organic mercury compounds are more toxic than inorganic compounds because they are readily absorbed from the gastrointestinal tract (95%) and distributed throughout the body.^14^ Nevertheless, all forms of mercury are toxic and have been associated with neurological damage, renal dysfunction, gastrointestinal ulceration, and hepatotoxicity in humans.^14^ ^,^ ^59^

The *sil* operon (silver resistance) was only identified in the Tunapuco. The natural sources of silver in the environment include volcanic activity and hydrothermal-sedimentary deposits.^60^ In contrast, anthropogenic sources of environmental Ag pollution include soil pollution, coal and cement plants, metallurgy, waste landfills, sludge fertilisers, and silver nanoparticle combustion.^60^ Silver is generally not considered carcinogenic or toxic to the immune, cardiovascular, nervous, or reproductive systems.^61^ However, overexposure can lead to acute symptoms such as low blood pressure, diarrhoea, stomach irritation, and reduced respiration. Prolonged intake can affect the liver and kidneys, alter blood cells, and cause argyria and/or argyrosis. Moreover, small amounts of soluble silver compounds can accumulate in the brain and muscles.^61^

Both groups harbour proteins associated with copper resistance; however, the *pco* system was only identified in the Tunapuco group. This system detoxifies copper in the periplasm and relies on the activity of *copA* to confer resistance.^62^ The copper systems identified in both groups were *cue* and *cus*, both of which catalyse the removal of excess copper from the cells.^44^ The former system contains *cueR*, which regulates the expression of *cueO* and *copA*.^62^ The *copA* extrudes the excess copper from the cytoplasm, while *cueO*, as well as the *cus* system, are involved in periplasmic copper detoxification.^62^ Additionally, the *cus* system can be activated by silver, but higher concentrations of silver are required compared to those observed for copper.^44^

Other prevalent proteins identified in the Yanomami microbiome are CHRA1 and PSTB. The former reduces chromate accumulation in cells, and the latter is involved in phosphate import and arsenic resistance. Bioaccumulation of both metals in the human body can cause dermal, renal, neurological, and gastrointestinal diseases and several cancers. Arsenic, however, is of particular concern because it can endanger health even at very low concentrations and affect the cardiovascular, hepato-biliary, and respiratory systems, in addition to those already mentioned.^14^ ^,^ ^63^ ^,^ ^64^ ^,^ ^65^ In the Amazon Forest, chromium concentrations in a pristine area have been reported to be higher than the background concentration, but its origin was presumed to be geogenic.^65^ A study from 2005 reported that arsenic concentrations in Amazon rivers were higher in those flowing from the Andes, which is not the case for rivers to which the Yanomami have access.^66^ Since then, however, gold mining in the Yanomami region has increased substantially,^26^ and elevated arsenic concentrations in sediments, soils, and water in other regions of Brazil have been linked to various anthropogenic activities.^67^

For decades, the Yanomami ecological niche has been under the continuous discharge of batteries that also contributes to releasing metals into their environment.^30^ The most commonly used battery types contain significant amounts of heavy metals whose resistance genes were identified in our study, such as cobalt, copper, and nickel.^68^ Interestingly, cobalt and copper, as well as zinc, are essential for human life. However, they can also be toxic when present in excess.^2^

The proteins *arsR*, *cutA*, *yhcN*, and *zur* had the largest number of copies in the Tunapuco. Each protein represents a distinct metal resistance mechanism, with *arsR* being a transcriptional regulator associated with arsenic resistance,^43^ *cutA* being potentially involved in copper tolerance,^69^ *yhcN* being a stress-response protein associated with cadmium,^70^ and *zur* being a regulator of zinc uptake.^47^

We noticed a pattern in the metagenomes, considering different metal operons. Specifically, metagenomes containing genes with higher RPKM abundances usually presented all genes belonging to each operon. In contrast, most genes with lower RPKM abundance were the sole representatives of their respective operons. Therefore, we hypothesise that microbiomes in which operon genes exhibit the highest RPKM abundances are likely expressing the resistance phenotype.

As for the bacteria carrying these MR genes, most contigs were classified as *Proteobacteria*. This phylum is widespread and ubiquitous, and due to its adaptability and tolerance, it has been reported to harbour a variety of MR determinants in different environments and contexts.^2^ Interestingly, we have previously observed that the gut microbiome of Yanomami individuals differs from that of other semi-isolated human groups in the high abundance of *Proteobacteria*.^30^ However, it is not known whether this is the reason why these individuals have more *Proteobacteria* in their gut microbiome. In fact, we have previously linked this high abundance to the high exposure of the Yanomami to solar UVB light.^71^ However, it is also worth considering that most contigs were classified as *Proteobacteria*, or specifically as *E. coli* due to the over-representation of *E. coli* genomes in databases. It is a fact that *E. coli* strains are the most commonly used prokaryote model in studies related to resistance or virulence genes. Therefore, the classification of contigs harbouring metal resistance genes may be biased, as their genetic content comprises genes typically found in *E. coli*.

In summary, identifying genes that encode proteins associated with metal resistance in the faeces of Yanomami and Tunapuco individuals suggests a high abundance of heavy metals in the ecological niche they explore. Heavy metals from geological and anthropogenic sources are both widespread in the environment and non-biodegradable, which may act as long-term selection pressures.^1^ Therefore, we cannot trace the source or time period of exposure with this study, but the evidence shows that increases in gold mining have direct impacts on the Yanomami group, as do increases in deforestation rates.^20^ ^,^ ^26^ Monitoring and remediation of these activities are urgently important not only for the health and survival of indigenous groups but also for the conservation of the Amazon Region overall, which implies preserving global biodiversity and regulating the climate and hydrological cycle.^72^
