# Supplementary material for: Heavy metal resistance in the Yanomami and Tunapuco microbiome
Source: Mem Inst Oswaldo Cruz. 2023 Nov 10;118:e230086. doi: 10.1590/0074-02760230086 (PMC10641926; doi:10.1590/0074-02760230086)
Supplement: Supplementary file 1 [file 1678-8060-mioc-118-e230086-s.pdf]

TABLE I

Metal resistance proteins identified in the metagenomic contigs assembled from the Yanomami gut microbiome

Sample	Contig ID	gene ID	BacMet ID	Compound	Contig taxonomic classification
AH08	NODE_14158_length_709_cov_0.121993	merP	BAC0231	Mercury (Hg)	*Burkholderia cepacia*
AH08	NODE_24_length_48153_cov_1.061966	copR	BAC0719	Copper (Cu)	*Ralstonia pickettii*
AH08	NODE_2583_length_4457_cov_0.135797	merR	BAC0232	Mercury (Hg)	*Aeromonas salmonicida*
AH08	NODE_2583_length_4457_cov_0.135797	merT	BAC0233	Mercury (Hg)	*Aeromonas salmonicida*
AH08	NODE_2583_length_4457_cov_0.135797	merP	BAC0678	Mercury (Hg)	*Aeromonas salmonicida*
AH08	NODE_2669_length_4278_cov_0.238015	merA	BAC0652	Mercury (Hg)	*Aeromonas salmonicida*
AH08	NODE_2669_length_4278_cov_0.238015	merE	BAC0670	Mercury (Hg)	*Aeromonas salmonicida*
AH08	NODE_2669_length_4278_cov_0.238015	merD	BAC0668	Mercury (Hg)	*Aeromonas salmonicida*
AH08	NODE_276_length_18487_cov_0.677397	chrA1	BAC0548	Chromium (Cr)	*Ralstonia solanacearum*
AH08	NODE_332_length_16943_cov_0.349370	chrC	BAC0547	Chromium (Cr)	*Ralstonia mannitolilytica*
AH08	NODE_332_length_16943_cov_0.349370	chrA1	BAC0548	Chromium (Cr)	*Ralstonia mannitolilytica*
AH08	NODE_332_length_16943_cov_0.349370	cnrA	BAC0203	Cobalt (Co), Nickel (Ni)	*Ralstonia mannitolilytica*
AH08	NODE_332_length_16943_cov_0.349370	cnrR/cnrX	BAC0291	Cobalt (Co), Nickel (Ni)	*Ralstonia mannitolilytica*
AH08	NODE_785_length_11206_cov_0.220056	pstB	BAC0316	Arsenic (As)	*Paraburkholderia xenovorans*
AH08	NODE_9967_length_972_cov_0.099408	merR2	BAC0688	Mercury (Hg)	*Burkholderiales*
AH19	NODE_1440_length_2036_cov_1.150864	znuB/yebI	BAC0464	Zinc (Zn)	*Escherichia coli*
AH19	NODE_2310_length_1521_cov_0.751076	yhcN	BAC0446	Cadmium (Cd)	*Escherichia coli*
AH19	NODE_322_length_5063_cov_1.341775	corA	BAC0086	Magnesium (Mg), Cobalt (Co), Nickel (Ni), Manganese (Mn)	*Escherichia coli*
AH19	NODE_5692_length_896_cov_0.994798	cutA	BAC0113	Copper (Cu)	*Enterobacteriaceae*
AH19	NODE_7491_length_753_cov_1.119808	rcnB/yohN	BAC0331	Nickel (Ni), Cobalt (Co)	*Escherichia coli*
AH19	NODE_8237_length_709_cov_0.853952	dsbA	BAC0136	Cadmium (Cd), Zinc (Zn), Mercury (Hg)	*Escherichia coli*
AH19	NODE_9054_length_669_cov_1.001845	soxS	BAC0371	Zinc (Zn)	*Escherichia coli*
AL18	NODE_10376_length_1130_cov_0.102692	zntR/yhdM	BAC0462	Zinc (Zn)	*Escherichia coli*
AL18	NODE_10875_length_1105_cov_0.137014	yqjH	BAC0452	Iron (Fe), Nickel (Ni)	*Enterobacteriaceae*
AL18	NODE_1527_length_3000_cov_0.441351	rcnB/yohN	BAC0331	Nickel (Ni), Cobalt (Co)	*Escherichia coli*
AL18	NODE_18080_length_848_cov_0.070735	ychH	BAC0434	Cadmium (Cd)	*Escherichia coli*
AL18	NODE_2291_length_2438_cov_0.282994	arsC	BAC0584	Arsenic (As), Antimony (Sb)	*Escherichia coli*
AL18	NODE_23790_length_730_cov_0.054726	dsbB	BAC0137	Cadmium (Cd), Mercury (Hg)	*Klebsiella*
AL18	NODE_32336_length_612_cov_0.117526	mntR	BAC0253	Manganese (Mn), Magnesium (Mg)	*Citrobacter*
AL18	NODE_3499_length_1969_cov_0.458740	zinT/yodA	BAC0457	Cadmium (Cd), Zinc (Zn)	*Escherichia coli*
AL18	NODE_4478_length_1730_cov_0.110418	fecD	BAC0163	Nickel (Ni), Cobalt (Co)	*Escherichia coli*
AL18	NODE_5044_length_1637_cov_0.184106	znuC/yebM	BAC0465	Zinc (Zn)	*Escherichia coli*
AL18	NODE_5044_length_1637_cov_0.184106	znuB/yebI	BAC0464	Zinc (Zn)	*Escherichia coli*
AL18	NODE_62554_length_414_cov_0.024390	merP	BAC0678	Mercury (Hg)	*Aeromonas salmonicida*
AL18	NODE_7789_length_1309_cov_0.082910	tehB	BAC0385	Tellurium (Te)	*Enterobacteriaceae*
AL18	NODE_8522_length_1249_cov_0.119430	modE	BAC0608	Tungsten (W), Molybdenum (Mo)	*Escherichia coli*
AL18	NODE_8871_length_1222_cov_0.667580	zitB/ybgR	BAC0459	Zinc (Zn)	*Enterobacteriaceae*
AL18	NODE_8957_length_1216_cov_0.121212	yfeB	BAC0440	Iron (Fe), Manganese (Mn)	*Vibrio hyugaensis*
AL19	NODE_102764_length_326_cov_0.065327	rcnR/yohL	BAC0332	Cobalt (Co), Nickel (Ni), Iron (Fe)	*Escherichia coli*
AL19	NODE_107_length_18611_cov_0.200931	chrC	BAC0547	Chromium (Cr)	*Ralstonia mannitolilytica*
AL19	NODE_107_length_18611_cov_0.200931	chrA1	BAC0548	Chromium (Cr)	*Ralstonia mannitolilytica*
AL19	NODE_107_length_18611_cov_0.200931	cnrA	BAC0203	Cobalt (Co), Nickel (Ni)	*Ralstonia mannitolilytica*
AL19	NODE_126517_length_287_cov_0.062500	merT	BAC0690	Mercury (Hg)	*Rhodanobacter denitrificans*
AL19	NODE_14874_length_991_cov_0.094907	merT	BAC0233	Mercury (Hg)	*Aeromonas salmonicida*
AL19	NODE_14874_length_991_cov_0.094907	merP	BAC0678	Mercury (Hg)	*Aeromonas salmonicida*
AL19	NODE_15_length_48368_cov_0.383927	copR	BAC0719	Copper (Cu)	*Ralstonia pickettii*

Sample	Contig ID	gene ID	BacMet ID	Compound	Contig taxonomic classification
AL19	NODE_1904_length_3073_cov_0.153428	merE	BAC0670	Mercury (Hg)	*Halothiobacillus neapolitanus*
AL19	NODE_1904_length_3073_cov_0.153428	merD	BAC0668	Mercury (Hg)	*Halothiobacillus neapolitanus*
AL19	NODE_23166_length_792_cov_0.076692	merT	BAC0690	Mercury (Hg)	*Paraburkholderia fungorum*
AL19	NODE_251_length_11094_cov_0.384426	pstB	BAC0316	Arsenic (As)	*Kinneretia sp. DAIF2*
AL19	NODE_25575_length_752_cov_0.072000	merR2	BAC0688	Mercury (Hg)	*Proteobacteria*
AL19	NODE_28038_length_716_cov_0.062818	copC	BAC0639	Copper (Cu)	*Pseudomonas sp. So3.2b*
AL19	NODE_4700_length_1806_cov_0.141751	cnrR/cnrX	BAC0291	Cobalt (Co), Nickel (Ni)	*Ralstonia mannitolilytica*
AL19	NODE_5724_length_1623_cov_0.167112	rcnB/yohN	BAC0331	Nickel (Ni), Cobalt (Co)	*Escherichia coli*
AL19	NODE_6962_length_1466_cov_0.147125	zitB/ybgR	BAC0459	Zinc (Zn)	*Escherichia coli*
AL19	NODE_806_length_5312_cov_0.148891	pstB	BAC0316	Arsenic (As)	*Ralstonia solanacearum*
AL19	NODE_8596_length_1314_cov_0.126369	zur/yjbK	BAC0470	Zinc (Zn)	*Escherichia coli*
AL19	NODE_92937_length_347_cov_0.104545	ychH	BAC0434	Cadmium (Cd)	*Enterobacteriaceae*
AL19	NODE_94_length_19670_cov_0.518651	chrA1	BAC0548	Chromium (Cr)	*Ralstonia solanacearum*
CA02	NODE_5135_length_477_cov_0.737143	zur/yjbK	BAC0470	Zinc (Zn)	*Enterobacteriaceae*
CA09	NODE_7658_length_861_cov_0.347411	dsbA	BAC0136	Cadmium (Cd), Zinc (Zn), Mercury (Hg)	*Escherichia coli*
CA46	NODE_10_length_157304_cov_4.338733	soxS	BAC0371	Zinc (Zn)	*Escherichia coli*
CA46	NODE_10_length_157304_cov_4.338733	zur/yjbK	BAC0470	Zinc (Zn)	*Escherichia coli*
CA46	NODE_116_length_31037_cov_5.168540	zraR/hydH	BAC0467	Zinc (Zn)	*Escherichia coli*
CA46	NODE_116_length_31037_cov_5.168540	zraS/hydG	BAC0468	Zinc (Zn), Lead (Pb)	*Escherichia coli*
CA46	NODE_126_length_28059_cov_4.351762	modC	BAC0611	Tungsten (W), Molybdenum (Mo)	*Escherichia coli*
CA46	NODE_126_length_28059_cov_4.351762	modE	BAC0608	Tungsten (W), Molybdenum (Mo)	*Escherichia coli*
CA46	NODE_126_length_28059_cov_4.351762	modB	BAC0610	Tungsten (W), Molybdenum (Mo)	*Escherichia coli*
CA46	NODE_126_length_28059_cov_4.351762	modA	BAC0609	Tungsten (W), Molybdenum (Mo)	*Escherichia coli*
CA46	NODE_126_length_28059_cov_4.351762	zitB/ybgR	BAC0459	Zinc (Zn)	*Escherichia coli*
CA46	NODE_133_length_26226_cov_6.397491	zntR/yhdM	BAC0462	Zinc (Zn)	*Escherichia coli*
CA46	NODE_13_length_147547_cov_4.105384	baeS	BAC0040	Zinc (Zn), Tungsten (W)	*Escherichia coli*
CA46	NODE_13_length_147547_cov_4.105384	rcnR/yohL	BAC0332	Cobalt (Co), Nickel (Ni), Iron (Fe)	*Escherichia coli*
CA46	NODE_13_length_147547_cov_4.105384	rcnB/yohN	BAC0331	Nickel (Ni), Cobalt (Co)	*Escherichia coli*
CA46	NODE_13_length_147547_cov_4.105384	baeR	BAC0039	Zinc (Zn), Tungsten (W)	*Escherichia coli*
CA46	NODE_13_length_147547_cov_4.105384	rcnA/yohM	BAC0330	Cobalt (Co), Nickel (Ni), Iron (Fe)	*Escherichia coli*
CA46	NODE_14_length_136364_cov_4.585360	dsbC	BAC0138	Copper (Cu)	*Escherichia coli*
CA46	NODE_15_length_127405_cov_4.621968	cueO	BAC0103	Copper (Cu)	*Escherichia coli*
CA46	NODE_15_length_127405_cov_4.621968	cutF/nlpE	BAC0116	Copper (Cu)	*Escherichia coli*
CA46	NODE_200_length_18929_cov_4.625610	glpF	BAC0181	Antimony (Sb), Arsenic (As)	*Escherichia coli*
CA46	NODE_22_length_92922_cov_4.215973	corD	BAC0644	Cobalt (Co), Magnesium (Mg)	*Escherichia coli*
CA46	NODE_25_length_88626_cov_4.378435	bhsA/ycfR/comC	BAC0049	Copper (Cu)	*Escherichia coli*
CA46	NODE_25_length_88626_cov_4.378435	comR/ycfQ	BAC0076	Copper (Cu)	*Escherichia coli*
CA46	NODE_261_length_15498_cov_3.444264	cusR/ylcA	BAC0111	Copper (Cu), Silver (Ag)	*Escherichia coli*
CA46	NODE_261_length_15498_cov_3.444264	cusA/ybdE	BAC0107	Copper (Cu), Silver (Ag)	*Escherichia coli*
CA46	NODE_261_length_15498_cov_3.444264	cusC/ylcB	BAC0109	Copper (Cu), Silver (Ag)	*Escherichia coli*
CA46	NODE_261_length_15498_cov_3.444264	cusF/cusX	BAC0110	Copper (Cu), Silver (Ag)	*Escherichia coli*
CA46	NODE_261_length_15498_cov_3.444264	cusB	BAC0108	Copper (Cu), Silver (Ag)	*Escherichia coli*
CA46	NODE_261_length_15498_cov_3.444264	cusS	BAC0112	Copper (Cu), Silver (Ag)	*Escherichia coli*
CA46	NODE_26_length_88493_cov_4.762848	corA	BAC0086	Magnesium (Mg), Cobalt (Co), Nickel (Ni), Manganese (Mn)	*Escherichia coli*
CA46	NODE_27_length_82884_cov_4.662806	sodB	BAC0707	Selenium (Se)	*Escherichia coli*
CA46	NODE_28_length_81756_cov_4.326008	dsbB	BAC0137	Cadmium (Cd), Mercury (Hg)	*Escherichia coli*
CA46	NODE_28_length_81756_cov_4.326008	ychH	BAC0434	Cadmium (Cd)	*Escherichia coli*
CA46	NODE_32_length_74669_cov_4.376327	znuC/yebM	BAC0465	Zinc (Zn)	*Escherichia coli*
CA46	NODE_32_length_74669_cov_4.376327	znuA/yebL	BAC0463	Zinc (Zn)	*Escherichia coli*

Sample	Contig ID	gene ID	BacMet ID	Compound	Contig taxonomic classification
CA46	NODE_32_length_74669_cov_4.376327	znuB/yebI	BAC0464	Zinc (Zn)	*Escherichia coli*
CA46	NODE_32_length_74669_cov_4.376327	mntP/yebN	BAC0252	Manganese (Mn), Magnesium (Mg)	*Escherichia coli*
CA46	NODE_35_length_69249_cov_4.359857	sodA	BAC0368	Selenium (Se)	*Escherichia coli*
CA46	NODE_35_length_69249_cov_4.359857	dsbA	BAC0136	Cadmium (Cd), Zinc (Zn), Mercury (Hg)	*Escherichia coli*
CA46	NODE_35_length_69249_cov_4.359857	fieF/yiip	BAC0167	Iron (Fe), Zinc (Zn), Cobalt (Co), Cadmium (Cd), Nickel (Ni)	*Escherichia coli*
CA46	NODE_39_length_68095_cov_4.534079	corB	BAC0643	Cobalt (Co), Magnesium (Mg)	*Escherichia coli*
CA46	NODE_3_length_199565_cov_4.663940	pstA	BAC0315	Arsenic (As)	*Escherichia coli*
CA46	NODE_3_length_199565_cov_4.663940	pstB	BAC0316	Arsenic (As)	*Escherichia coli*
CA46	NODE_3_length_199565_cov_4.663940	pstS	BAC0318	Arsenic (As)	*Escherichia coli*
CA46	NODE_3_length_199565_cov_4.663940	yieF	BAC0541	Chromium (Cr), Vanadium (V), Molybdenum (Mo)	*Escherichia coli*
CA46	NODE_3_length_199565_cov_4.663940	pstC	BAC0317	Arsenic (As)	*Escherichia coli*
CA46	NODE_43_length_65748_cov_5.060057	yhcN	BAC0446	Cadmium (Cd)	*Escherichia coli*
CA46	NODE_4_length_196382_cov_4.412088	tehA	BAC0384	Tellurium (Te)	*Escherichia coli*
CA46	NODE_4_length_196382_cov_4.412088	tehB	BAC0385	Tellurium (Te)	*Escherichia coli*
CA46	NODE_52_length_58742_cov_4.810193	nfsA	BAC0540	Chromium (Cr)	*Escherichia coli*
CA46	NODE_56_length_57198_cov_5.031302	arsC	BAC0584	Arsenic (As), Antimony (Sb)	*Escherichia coli*
CA46	NODE_56_length_57198_cov_5.031302	nikR	BAC0275	Nickel (Ni)	*Escherichia coli*
CA46	NODE_56_length_57198_cov_5.031302	nikC	BAC0272	Nickel (Ni)	*Escherichia coli*
CA46	NODE_56_length_57198_cov_5.031302	pitA	BAC0312	Zinc (Zn), Tellurium (Te)	*Escherichia coli*
CA46	NODE_56_length_57198_cov_5.031302	nikA	BAC0270	Nickel (Ni)	*Escherichia coli*
CA46	NODE_56_length_57198_cov_5.031302	nikB	BAC0271	Nickel (Ni)	*Escherichia coli*
CA46	NODE_56_length_57198_cov_5.031302	nikD	BAC0273	Nickel (Ni)	*Escherichia coli*
CA46	NODE_56_length_57198_cov_5.031302	nikE	BAC0274	Nickel (Ni)	*Escherichia coli*
CA46	NODE_56_length_57198_cov_5.031302	arsR	BAC0594	Arsenic (As)	*Escherichia coli*
CA46	NODE_56_length_57198_cov_5.031302	zntA/yhhO	BAC0461	Lead (Pb), Cadmium (Cd), Zinc (Zn)	*Escherichia coli*
CA46	NODE_56_length_57198_cov_5.031302	arsB	BAC0576	Arsenic (As), Antimony (Sb)	*Escherichia coli*
CA46	NODE_60_length_53974_cov_4.629478	cutC	BAC0114	Copper (Cu)	*Escherichia coli*
CA46	NODE_61_length_53135_cov_4.293999	mntR	BAC0253	Manganese (Mn), Magnesium (Mg)	*Escherichia coli*
CA46	NODE_6_length_175989_cov_4.182472	zupT/ygiE	BAC0469	Zinc (Zn), Iron (Fe), Cobalt (Co), Nickel (Ni), Copper (Cu), Cadmium (Cd)	*Escherichia coli*
CA46	NODE_6_length_175989_cov_4.182472	ygiW	BAC0445	Cadmium (Cd)	*Escherichia coli*
CA46	NODE_6_length_175989_cov_4.182472	pitA	BAC0312	Zinc (Zn), Tellurium (Te)	*Escherichia coli*
CA46	NODE_6_length_175989_cov_4.182472	yqjH	BAC0452	Iron (Fe), Nickel (Ni)	*Escherichia coli*
CA46	NODE_70_length_47761_cov_4.281122	corC	BAC0088	Cobalt (Co), Magnesium (Mg)	*Escherichia coli*
CA46	NODE_70_length_47761_cov_4.281122	cutE/lnt	BAC0115	Copper (Cu)	*Escherichia coli*
CA46	NODE_76_length_44860_cov_4.022576	mntH/yfeP	BAC0251	Manganese (Mn), Iron (Fe), Cadmium (Cd), Cobalt (Co), Zinc (Zn)	*Escherichia coli*
CA46	NODE_7_length_168873_cov_4.660140	mgtA	BAC0087	Cobalt (Co), Magnesium (Mg)	*Escherichia coli*
CA46	NODE_80_length_42033_cov_4.565879	robA	BAC0334	Silver (Ag), Mercury (Hg), Cadmium (Cd)	*Escherichia coli*
CA46	NODE_8_length_167646_cov_4.342939	cueR/ybbI	BAC0105	Copper (Cu)	*Escherichia coli*
CA46	NODE_8_length_167646_cov_4.342939	fetA/ybbL	BAC0165	Iron (Fe)	*Escherichia coli*
CA46	NODE_8_length_167646_cov_4.342939	fetB/ybbM	BAC0166	Iron (Fe)	*Escherichia coli*
CA46	NODE_8_length_167646_cov_4.342939	copA	BAC0725	Copper (Cu)	*Escherichia coli*
CA46	NODE_90_length_38962_cov_3.977755	yodD	BAC0451	Cadmium (Cd)	*Escherichia coli*
CA46	NODE_90_length_38962_cov_3.977755	zinT/yodA	BAC0457	Cadmium (Cd), Zinc (Zn)	*Escherichia coli*
CA60	NODE_8819_length_395_cov_0.052239	chrF	BAC0066	Chromium (Cr)	*Ralstonia mannitolilytica*
TA08	NODE_10037_length_2136_cov_4.084121	ygiW	BAC0445	Cadmium (Cd)	*Escherichia coli*
TA08	NODE_10185_length_2109_cov_4.481332	zitB/ybgR	BAC0459	Zinc (Zn)	*Enterobacteriaceae*
TA08	NODE_10856_length_2007_cov_3.997872	arsR	BAC0594	Arsenic (As)	*Escherichia coli*

Sample	Contig ID	gene ID	BacMet ID	Compound	Contig taxonomic classification
TA08	NODE_10856_length_2007_cov_3.997872	arsB	BAC0576	Arsenic (As), Antimony (Sb)	*Escherichia coli*
TA08	NODE_1101_length_12088_cov_4.426219	copB	BAC0079	Copper (Cu), Silver (Ag)	*Enterococcus hirae*
TA08	NODE_11583_length_1915_cov_5.294743	soxS	BAC0371	Zinc (Zn)	*Escherichia coli*
TA08	NODE_1165_length_11551_cov_4.499475	cutA	BAC0113	Copper (Cu)	*Escherichia coli*
TA08	NODE_12481_length_1812_cov_2.944807	bhsA/ycfR/comC	BAC0049	Copper (Cu)	*Escherichia coli*
TA08	NODE_12481_length_1812_cov_2.944807	comR/ycfQ	BAC0076	Copper (Cu)	*Escherichia coli*
TA08	NODE_1267_length_10673_cov_3.869334	glpF	BAC0181	Antimony (Sb), Arsenic (As)	*Escherichia coli*
TA08	NODE_12741_length_1785_cov_3.247889	arsR	BAC0594	Arsenic (As)	*Escherichia coli*
TA08	NODE_13131_length_1746_cov_0.962322	cusF/cusX	BAC0110	Copper (Cu), Silver (Ag)	*Escherichia coli*
TA08	NODE_13131_length_1746_cov_0.962322	cusB	BAC0108	Copper (Cu), Silver (Ag)	*Escherichia coli*
TA08	NODE_1334_length_10182_cov_5.195027	tehA	BAC0384	Tellurium (Te)	*Escherichia coli*
TA08	NODE_1334_length_10182_cov_5.195027	tehB	BAC0385	Tellurium (Te)	*Escherichia coli*
TA08	NODE_1407_length_9776_cov_4.328013	yhcN	BAC0446	Cadmium (Cd)	*Escherichia coli*
TA08	NODE_14629_length_1617_cov_1.787248	pstA	BAC0315	Arsenic (As)	*Escherichia coli*
TA08	NODE_15699_length_1535_cov_19.911932	rcnB/yohN	BAC0331	Nickel (Ni), Cobalt (Co)	*Escherichia coli*
TA08	NODE_16294_length_1495_cov_6.097953	pstB	BAC0316	Arsenic (As)	*Enterobacteriaceae*
TA08	NODE_1656_length_8636_cov_6.212128	dsbB	BAC0137	Cadmium (Cd), Mercury (Hg)	*Escherichia coli*
TA08	NODE_1736_length_8335_cov_3.040570	zntR/yhdM	BAC0462	Zinc (Zn)	*Escherichia coli*
TA08	NODE_17584_length_1413_cov_2.528771	pstS	BAC0318	Arsenic (As)	*Escherichia coli*
TA08	NODE_1785_length_8129_cov_3.233567	sodB	BAC0707	Selenium (Se)	*Escherichia coli*
TA08	NODE_1888_length_7808_cov_2.625700	ychH	BAC0434	Cadmium (Cd)	*Escherichia coli*
TA08	NODE_2119_length_7121_cov_10.377466	dsbA	BAC0136	Cadmium (Cd), Zinc (Zn), Mercury (Hg)	*Escherichia coli*
TA08	NODE_2219_length_6849_cov_7.245463	sitC	BAC0351	Manganese (Mn), Iron (Fe)	*Escherichia coli*
TA08	NODE_2276_length_6712_cov_2.680030	zraS/hydG	BAC0468	Zinc (Zn), Lead (Pb)	*Escherichia coli*
TA08	NODE_22_length_137162_cov_6.713737	copB	BAC0079	Copper (Cu), Silver (Ag)	*Enterococcus mundtii QU 25*
TA08	NODE_23421_length_1146_cov_1.061825	nikR	BAC0275	Nickel (Ni)	*Escherichia coli*
TA08	NODE_2342_length_6570_cov_2.815459	yqjH	BAC0452	Iron (Fe), Nickel (Ni)	*Escherichia coli*
TA08	NODE_24178_length_1118_cov_1.775984	nikC	BAC0272	Nickel (Ni)	*Escherichia coli*
TA08	NODE_2610_length_6031_cov_2.924797	corA	BAC0086	Magnesium (Mg), Cobalt (Co), Nickel (Ni), Manganese (Mn)	*Escherichia coli*
TA08	NODE_2624_length_6005_cov_5.264886	nfsA	BAC0540	Chromium (Cr)	*Escherichia coli*
TA08	NODE_2725_length_5829_cov_4.122939	pitA	BAC0312	Zinc (Zn), Tellurium (Te)	*Escherichia coli*
TA08	NODE_2772_length_5762_cov_3.926886	sodA	BAC0368	Selenium (Se)	*Escherichia coli*
TA08	NODE_2825_length_5693_cov_2.993532	modC	BAC0611	Tungsten (W), Molybdenum (Mo)	*Escherichia coli*
TA08	NODE_2825_length_5693_cov_2.993532	modB	BAC0610	Tungsten (W), Molybdenum (Mo)	*Escherichia coli*
TA08	NODE_2825_length_5693_cov_2.993532	modA	BAC0609	Tungsten (W), Molybdenum (Mo)	*Escherichia coli*
TA08	NODE_2829_length_5686_cov_3.368951	cueO	BAC0103	Copper (Cu)	*Escherichia coli*
TA08	NODE_2830_length_5685_cov_5.464556	znuC/yebM	BAC0465	Zinc (Zn)	*Escherichia coli*
TA08	NODE_2830_length_5685_cov_5.464556	znuA/yebL	BAC0463	Zinc (Zn)	*Escherichia coli*
TA08	NODE_2929_length_5563_cov_3.362951	robA	BAC0334	Silver (Ag), Mercury (Hg), Cadmium (Cd)	*Escherichia coli*
TA08	NODE_3003_length_5445_cov_2.379278	corB	BAC0643	Cobalt (Co), Magnesium (Mg)	*Escherichia albertii*
TA08	NODE_3019_length_5425_cov_3.038694	cusA/ybdE	BAC0107	Copper (Cu), Silver (Ag)	*Escherichia coli*
TA08	NODE_3634_length_4681_cov_4.594422	mntH/yfeP	BAC0251	Manganese (Mn), Iron (Fe), Cadmium (Cd), Cobalt (Co), Zinc (Zn)	*Escherichia coli*
TA08	NODE_38542_length_798_cov_5.697466	rcnA/yohM	BAC0330	Cobalt (Co), Nickel (Ni), Iron (Fe)	*Escherichia coli*
TA08	NODE_38543_length_798_cov_5.533532	rcnA/yohM	BAC0330	Cobalt (Co), Nickel (Ni), Iron (Fe)	*Escherichia coli*
TA08	NODE_4077_length_4296_cov_2.624610	cutC	BAC0114	Copper (Cu)	*Escherichia coli*
TA08	NODE_4101_length_4277_cov_5.902892	pitA	BAC0312	Zinc (Zn), Tellurium (Te)	*Escherichia coli*
TA08	NODE_4165_length_4224_cov_2.748108	nikA	BAC0270	Nickel (Ni)	*Escherichia coli*
TA08	NODE_4165_length_4224_cov_2.748108	nikB	BAC0271	Nickel (Ni)	*Escherichia coli*

Sample	Contig ID	gene ID	BacMet ID	Compound	Contig taxonomic classification
TA08	NODE_44503_length_718_cov_1.365482	cusR/ylcA	BAC0111	Copper (Cu), Silver (Ag)	*Escherichia coli*
TA08	NODE_451_length_26080_cov_9.414172	zinT/yodA	BAC0457	Cadmium (Cd), Zinc (Zn)	*Escherichia coli*
TA08	NODE_4594_length_3912_cov_2.315720	dsbC	BAC0138	Copper (Cu)	*Escherichia coli*
TA08	NODE_46970_length_691_cov_2.150709	corB	BAC0643	Cobalt (Co), Magnesium (Mg)	*Escherichia coli*
TA08	NODE_4711_length_3830_cov_5.159060	mgtA	BAC0087	Cobalt (Co), Magnesium (Mg)	*Escherichia coli*
TA08	NODE_4985_length_3654_cov_4.943011	fieF/yiip	BAC0167	Iron (Fe), Zinc (Zn), Cobalt (Co), Cadmium (Cd), Nickel (Ni)	*Escherichia coli*
TA08	NODE_5120_length_3583_cov_8.217882	yodD	BAC0451	Cadmium (Cd)	*Escherichia coli*
TA08	NODE_5121_length_3582_cov_7.633575	zupT/ygiE	BAC0469	Zinc (Zn), Iron (Fe), Cobalt (Co), Nickel (Ni), Copper (Cu), Cadmium (Cd)	*Escherichia coli*
TA08	NODE_5302_length_3473_cov_3.592648	corD	BAC0644	Cobalt (Co), Magnesium (Mg)	*Escherichia coli*
TA08	NODE_56017_length_609_cov_1.842324	yjaA	BAC0447	Cadmium (Cd)	*Escherichia coli*
TA08	NODE_5632_length_3313_cov_3.241368	corC	BAC0088	Cobalt (Co), Magnesium (Mg)	*Escherichia coli*
TA08	NODE_5632_length_3313_cov_3.241368	cutE/lnt	BAC0115	Copper (Cu)	*Escherichia coli*
TA08	NODE_5821_length_3226_cov_4.470474	mntR	BAC0253	Manganese (Mn), Magnesium (Mg)	*Escherichia coli*
TA08	NODE_6366_length_3016_cov_4.233645	zur/yjbK	BAC0470	Zinc (Zn)	*Escherichia coli*
TA08	NODE_6521_length_2959_cov_2.788842	cueR/ybbI	BAC0105	Copper (Cu)	*Escherichia coli*
TA08	NODE_66175_length_541_cov_1.560386	cueR/ybbI	BAC0105	Copper (Cu)	*Escherichia coli*
TA08	NODE_7079_length_2787_cov_4.618797	yieF	BAC0541	Chromium (Cr), Vanadium (V), Molybdenum (Mo)	*Escherichia coli*
TA08	NODE_7242_length_2731_cov_6.375960	arsC	BAC0584	Arsenic (As), Antimony (Sb)	*Escherichia coli*
TA08	NODE_7841_length_2570_cov_7.142857	rcnR/yohL	BAC0332	Cobalt (Co), Nickel (Ni), Iron (Fe)	*Escherichia coli*
TA08	NODE_7925_length_2549_cov_4.217176	zinT/yodA	BAC0457	Cadmium (Cd), Zinc (Zn)	*Escherichia coli*
TA08	NODE_803_length_15869_cov_2.851798	copY/tcrY	BAC0084	Copper (Cu)	*Enterococcus hirae*
TA08	NODE_803_length_15869_cov_2.851798	copZ	BAC0085	Copper (Cu)	*Enterococcus hirae*
TA08	NODE_8703_length_2378_cov_4.729009	fetA/ybbL	BAC0165	Iron (Fe)	*Escherichia coli*
TA08	NODE_8703_length_2378_cov_4.729009	fetB/ybbM	BAC0166	Iron (Fe)	*Escherichia coli*
TA08	NODE_8800_length_2362_cov_2.128412	zntA/yhhO	BAC0461	Lead (Pb), Cadmium (Cd), Zinc (Zn)	*Escherichia coli*
TA08	NODE_9162_length_2289_cov_2.678538	baeR	BAC0039	Zinc (Zn), Tungsten (W)	*Escherichia coli*
TA24	NODE_11125_length_1510_cov_0.112798	merF	BAC0228	Mercury (Hg)	*Proteobacteria*
TA24	NODE_12308_length_1417_cov_0.269767	merT	BAC0695	Mercury (Hg)	*Gammaproteobacteria*
TA24	NODE_12308_length_1417_cov_0.269767	merR2	BAC0688	Mercury (Hg)	*Gammaproteobacteria*
TA24	NODE_169_length_26374_cov_0.909285	chrA1	BAC0548	Chromium (Cr)	*Ralstonia solanacearum*
TA24	NODE_1773_length_5130_cov_0.188287	czcC	BAC0121	Cadmium (Cd), Zinc (Zn), Cobalt (Co)	*Cupriavidus metallidurans*
TA24	NODE_18310_length_1114_cov_0.126646	merP	BAC0678	Mercury (Hg)	*Burkholderia*
TA24	NODE_18310_length_1114_cov_0.126646	merT	BAC0690	Mercury (Hg)	*Burkholderia*
TA24	NODE_18_length_101919_cov_0.530376	chrC	BAC0547	Chromium (Cr)	*Ralstonia mannitolilytica*
TA24	NODE_18_length_101919_cov_0.530376	chrA1	BAC0548	Chromium (Cr)	*Ralstonia mannitolilytica*
TA24	NODE_18_length_101919_cov_0.530376	cnrA	BAC0203	Cobalt (Co), Nickel (Ni)	*Ralstonia mannitolilytica*
TA24	NODE_18_length_101919_cov_0.530376	cnrR/cnrX	BAC0291	Cobalt (Co), Nickel (Ni)	*Ralstonia mannitolilytica*
TA24	NODE_20303_length_1047_cov_0.171739	merE	BAC0672	Mercury (Hg)	*Proteobacteria*
TA24	NODE_21_length_89528_cov_1.702240	copR	BAC0719	Copper (Cu)	*Ralstonia mannitolilytica*
TA24	NODE_237_length_20625_cov_0.309786	merT	BAC0690	Mercury (Hg)	*Paraburkholderia fungorum*
TA24	NODE_278_length_18494_cov_0.581042	merR	BAC0232	Mercury (Hg)	*Aeromonas salmonicida*
TA24	NODE_278_length_18494_cov_0.581042	merT	BAC0233	Mercury (Hg)	*Aeromonas salmonicida*
TA24	NODE_278_length_18494_cov_0.581042	merP	BAC0678	Mercury (Hg)	*Aeromonas salmonicida*
TA24	NODE_278_length_18494_cov_0.581042	merA	BAC0652	Mercury (Hg)	*Aeromonas salmonicida*
TA24	NODE_278_length_18494_cov_0.581042	merE	BAC0670	Mercury (Hg)	*Aeromonas salmonicida*
TA24	NODE_278_length_18494_cov_0.581042	merD	BAC0668	Mercury (Hg)	*Aeromonas salmonicida*
TA24	NODE_38382_length_703_cov_0.065972	cadX	BAC0059	Cadmium (Cd), Zinc (Zn)	*Streptococcus mitis B6*
TA24	NODE_41229_length_671_cov_0.091912	modB	BAC0610	Tungsten (W), Molybdenum (Mo)	*Escherichia coli*

Sample	Contig ID	gene ID	BacMet ID	Compound	Contig taxonomic classification
TA24	NODE_4298_length_2780_cov_0.545797	rcnB/yohN	BAC0331	Nickel (Ni), Cobalt (Co)	*Escherichia coli*
TA24	NODE_4298_length_2780_cov_0.545797	rcnA/yohM	BAC0330	Cobalt (Co), Nickel (Ni), Iron (Fe)	*Escherichia coli*
TA24	NODE_4373_length_2745_cov_0.147823	merR	BAC0232	Mercury (Hg)	*Paraburkholderia hospita*
TA24	NODE_56_length_53111_cov_0.470463	pstB	BAC0316	Arsenic (As)	*Ralstonia solanacearum*
TA24	NODE_6082_length_2197_cov_0.123671	merP	BAC0231	Mercury (Hg)	*Burkholderia cepacia*
TA24	NODE_6082_length_2197_cov_0.123671	merT	BAC0693	Mercury (Hg)	*Burkholderia cepacia*
TA24	NODE_6319_length_2138_cov_0.090502	zur/yjbK	BAC0470	Zinc (Zn)	*Escherichia coli*
TA24	NODE_999_length_7773_cov_0.216322	arsC	BAC0583	Arsenic (As), Antimony (Sb)	*Klebsiella pneumoniae*
TA91	NODE_1287_length_7938_cov_0.285623	chrC	BAC0547	Chromium (Cr)	*Ralstonia mannitolilytica*
TA91	NODE_1287_length_7938_cov_0.285623	chrA1	BAC0548	Chromium (Cr)	*Ralstonia mannitolilytica*
TA91	NODE_1287_length_7938_cov_0.285623	cnrA	BAC0203	Cobalt (Co), Nickel (Ni)	*Ralstonia mannitolilytica*
TA91	NODE_1287_length_7938_cov_0.285623	chrF	BAC0066	Chromium (Cr)	*Ralstonia mannitolilytica*
TA91	NODE_1520_length_7001_cov_0.194501	pstB	BAC0316	Arsenic (As)	*Ralstonia solanacearum*
TA91	NODE_15_length_57578_cov_2.213277	chrA1	BAC0548	Chromium (Cr)	*Ralstonia solanacearum*
TA91	NODE_2335_length_4701_cov_0.292742	cnrR/cnrX	BAC0291	Cobalt (Co), Nickel (Ni)	*Ralstonia mannitolilytica*
TA91	NODE_2431_length_4465_cov_0.231674	merA	BAC0652	Mercury (Hg)	*Aeromonas salmonicida*
TA91	NODE_2431_length_4465_cov_0.231674	merE	BAC0670	Mercury (Hg)	*Aeromonas salmonicida*
TA91	NODE_2431_length_4465_cov_0.231674	merD	BAC0668	Mercury (Hg)	*Aeromonas salmonicida*
TA91	NODE_4_length_71931_cov_1.491978	copR	BAC0719	Copper (Cu)	*Ralstonia mannitolilytica*
TA91	NODE_6169_length_1452_cov_0.164528	merR	BAC0232	Mercury (Hg)	*Aeromonas salmonicida*
TA91	NODE_6169_length_1452_cov_0.164528	merP	BAC0231	Mercury (Hg)	*Aeromonas salmonicida*
TA91	NODE_6169_length_1452_cov_0.164528	merT	BAC0233	Mercury (Hg)	*Aeromonas salmonicida*
TA91	NODE_6169_length_1452_cov_0.164528	merF	BAC0228	Mercury (Hg)	*Aeromonas salmonicida*
TA91	NODE_833_length_10484_cov_0.221010	pstB	BAC0316	Arsenic (As)	*Paraburkholderia xenovorans*
TA91	NODE_9619_length_970_cov_0.085409	merP	BAC0231	Mercury (Hg)	*Burkholderia cepacia*
TA91	NODE_9619_length_970_cov_0.085409	merT	BAC0693	Mercury (Hg)	*Burkholderia cepacia*

TABLE II

Metal resistance proteins identified in the metagenomic contigs assembled from the Tunapuco gut microbiome

Sample	Contig ID	gene ID	BacMet ID	Compound	Contig taxonomic classification
HCO72	NODE_10411_length_1492_cov_0.292308	nikD	BAC0273	Nickel (Ni)	*Enterobacteriaceae*
HCO64	NODE_110182_length_404_cov_0.184116	cusF/cusX	BAC0110	Copper (Cu), Silver (Ag)	*Escherichia coli*
HCO70	NODE_11088_length_2229_cov_0.484301	nikA	BAC0270	Nickel (Ni)	*Enterobacteriaceae*
HCO70	NODE_11122_length_2224_cov_0.749165	yhcN	BAC0446	Cadmium (Cd)	*Escherichia coli*
HCO72	NODE_11619_length_1397_cov_0.457480	nikB	BAC0271	Nickel (Ni)	*Escherichia*
HCO72	NODE_11772_length_1386_cov_0.433678	zur/yjbK	BAC0470	Zinc (Zn)	*Escherichia coli*
HCO70	NODE_12226_length_2100_cov_0.502788	znuC/yebM	BAC0465	Zinc (Zn)	*Escherichia coli*
HCO70	NODE_12226_length_2100_cov_0.502788	znuA/yebL	BAC0463	Zinc (Zn)	*Escherichia coli*
HCO70	NODE_12901_length_2029_cov_0.626183	ygiW	BAC0445	Cadmium (Cd)	*Escherichia coli*
HCO11	NODE_12_length_182646_cov_5.071286	nikR	BAC0275	Nickel (Ni)	*Escherichia coli*
HCO11	NODE_12_length_182646_cov_5.071286	nikC	BAC0272	Nickel (Ni)	*Escherichia coli*
HCO11	NODE_12_length_182646_cov_5.071286	nikA	BAC0270	Nickel (Ni)	*Escherichia coli*
HCO11	NODE_12_length_182646_cov_5.071286	nikB	BAC0271	Nickel (Ni)	*Escherichia coli*
HCO11	NODE_12_length_182646_cov_5.071286	nikD	BAC0273	Nickel (Ni)	*Escherichia coli*
HCO11	NODE_12_length_182646_cov_5.071286	nikE	BAC0274	Nickel (Ni)	*Escherichia coli*
HCO11	NODE_12_length_182646_cov_5.071286	zntA/yhhO	BAC0461	Lead (Pb), Cadmium (Cd), Zinc (Zn)	*Escherichia coli*
HCO11	NODE_12_length_182646_cov_5.071286	pcoA	BAC0303	Copper (Cu)	*Escherichia coli*
HCO11	NODE_12_length_182646_cov_5.071286	pcoB	BAC0304	Copper (Cu)	*Escherichia coli*

Sample	Contig ID	gene ID	BacMet ID	Compound	Contig taxonomic classification
HCO11	NODE_12_length_182646_cov_5.071286	pcoC	BAC0305	Copper (Cu)	*Escherichia coli*
HCO11	NODE_12_length_182646_cov_5.071286	pcoD	BAC0306	Copper (Cu)	*Escherichia coli*
HCO11	NODE_12_length_182646_cov_5.071286	pcoR	BAC0308	Copper (Cu)	*Escherichia coli*
HCO11	NODE_12_length_182646_cov_5.071286	pcoS	BAC0309	Copper (Cu)	*Escherichia coli*
HCO11	NODE_12_length_182646_cov_5.071286	pcoE	BAC0307	Copper (Cu), Silver (Ag)	*Escherichia coli*
HCO11	NODE_12_length_182646_cov_5.071286	silP	BAC0346	Silver (Ag)	*Escherichia coli*
HCO11	NODE_12_length_182646_cov_5.071286	silA	BAC0341	Silver (Ag)	*Escherichia coli*
HCO11	NODE_12_length_182646_cov_5.071286	silB	BAC0342	Silver (Ag)	*Escherichia coli*
HCO11	NODE_12_length_182646_cov_5.071286	silC	BAC0343	Silver (Ag)	*Escherichia coli*
HCO11	NODE_12_length_182646_cov_5.071286	silF	BAC0345	Silver (Ag)	*Escherichia coli*
HCO64	NODE_131020_length_361_cov_0.311966	soxS	BAC0371	Zinc (Zn)	*Escherichia coli*
HCO70	NODE_13998_length_1917_cov_0.458659	zraR/hydH	BAC0467	Zinc (Zn)	*Enterobacteriaceae*
HCO64	NODE_14348_length_1749_cov_0.380395	rcnB/yohN	BAC0331	Nickel (Ni), Cobalt (Co)	*Escherichia coli*
HCO70	NODE_14502_length_1870_cov_0.429719	rcnB/yohN	BAC0331	Nickel (Ni), Cobalt (Co)	*Escherichia coli*
HCO70	NODE_14634_length_1858_cov_0.430965	tehA	BAC0384	Tellurium (Te)	*Escherichia coli*
HCO72	NODE_14675_length_1212_cov_0.510599	mntR	BAC0253	Manganese (Mn), Magnesium (Mg)	*Escherichia albertii*
HCO70	NODE_14822_length_1842_cov_0.528863	cusR/ylcA	BAC0111	Copper (Cu), Silver (Ag)	*Escherichia*
HCO70	NODE_14824_length_1842_cov_0.453644	arsR	BAC0594	Arsenic (As)	*Escherichia*
HCO70	NODE_15344_length_1803_cov_0.375895	yqjH	BAC0452	Iron (Fe), Nickel (Ni)	*Escherichia coli*
HCO72	NODE_15988_length_1152_cov_0.459512	yhcN	BAC0446	Cadmium (Cd)	*Escherichia coli*
HCO72	NODE_16562_length_1128_cov_0.315684	bhsA/ycfR/ comC	BAC0049	Copper (Cu)	*Escherichia coli*
HCO11	NODE_16_length_148837_cov_5.023005	yhcN	BAC0446	Cadmium (Cd)	*Escherichia coli*
HCO70	NODE_17038_length_1684_cov_0.420681	yieF	BAC0541	Chromium (Cr), Vanadium (V), Molybdenum (Mo)	*Escherichia coli*
HCO64	NODE_17340_length_1546_cov_0.281184	dsbC	BAC0138	Copper (Cu)	*Escherichia coli*
HCO70	NODE_17402_length_1660_cov_0.729941	ychH	BAC0434	Cadmium (Cd)	*Escherichia coli*
HCO11	NODE_180_length_33307_cov_4.426522	arsC	BAC0584	Arsenic (As), Antimony (Sb)	*Escherichia coli*
HCO11	NODE_180_length_33307_cov_4.426522	pitA	BAC0312	Zinc (Zn), Tellurium (Te)	*Escherichia coli*
HCO11	NODE_180_length_33307_cov_4.426522	arsR	BAC0594	Arsenic (As)	*Escherichia coli*
HCO11	NODE_180_length_33307_cov_4.426522	arsB	BAC0576	Arsenic (As), Antimony (Sb)	*Escherichia coli*
HCO70	NODE_18436_length_1599_cov_0.538723	baeR	BAC0039	Zinc (Zn), Tungsten (W)	*Escherichia coli*
HCO11	NODE_186_length_31978_cov_4.159964	mntH/yfeP	BAC0251	Manganese (Mn), Iron (Fe), Cadmium (Cd), Cobalt (Co), Zinc (Zn)	*Escherichia coli*
HCO11	NODE_18_length_140309_cov_4.679773	zupT/ygiE	BAC0469	Zinc (Zn), Iron (Fe), Cobalt (Co), Nickel (Ni), Copper (Cu), Cadmium (Cd)	*Escherichia coli*
HCO11	NODE_18_length_140309_cov_4.679773	ygiW	BAC0445	Cadmium (Cd)	*Escherichia coli*
HCO11	NODE_18_length_140309_cov_4.679773	yqjH	BAC0452	Iron (Fe), Nickel (Ni)	*Escherichia coli*
HCO11	NODE_193_length_30938_cov_5.499367	zraR/hydH	BAC0467	Zinc (Zn)	*Escherichia coli*
HCO11	NODE_193_length_30938_cov_5.499367	zraS/hydG	BAC0468	Zinc (Zn), Lead (Pb)	*Escherichia coli*
HCO64	NODE_19477_length_1432_cov_0.327203	zntR/yhdM	BAC0462	Zinc (Zn)	*Enterobacteriaceae*
HCO11	NODE_19_length_135796_cov_4.335073	corC	BAC0088	Cobalt (Co), Magnesium (Mg)	*Escherichia coli*
HCO11	NODE_19_length_135796_cov_4.335073	cusR/ylcA	BAC0111	Copper (Cu), Silver (Ag)	*Escherichia coli*
HCO11	NODE_19_length_135796_cov_4.335073	cutE/lnt	BAC0115	Copper (Cu)	*Escherichia coli*
HCO11	NODE_19_length_135796_cov_4.335073	cusA/ybdE	BAC0107	Copper (Cu), Silver (Ag)	*Escherichia coli*
HCO11	NODE_19_length_135796_cov_4.335073	cusC/ylcB	BAC0109	Copper (Cu), Silver (Ag)	*Escherichia coli*
HCO11	NODE_19_length_135796_cov_4.335073	cusF/cusX	BAC0110	Copper (Cu), Silver (Ag)	*Escherichia coli*
HCO11	NODE_19_length_135796_cov_4.335073	cusB	BAC0108	Copper (Cu), Silver (Ag)	*Escherichia coli*
HCO11	NODE_19_length_135796_cov_4.335073	cusS	BAC0112	Copper (Cu), Silver (Ag)	*Escherichia coli*
HCO11	NODE_1_length_364039_cov_3.566728	znuC/yebM	BAC0465	Zinc (Zn)	*Escherichia coli*
HCO11	NODE_1_length_364039_cov_3.566728	sodB	BAC0707	Selenium (Se)	*Escherichia coli*

Sample	Contig ID	gene ID	BacMet ID	Compound	Contig taxonomic classification
HCO11	NODE_1_length_364039_cov_3.566728	znuA/yebL	BAC0463	Zinc (Zn)	*Escherichia coli*
HCO11	NODE_1_length_364039_cov_3.566728	znuB/yebI	BAC0464	Zinc (Zn)	*Escherichia coli*
HCO72	NODE_20065_length_1005_cov_0.323462	fetA/ybbL	BAC0165	Iron (Fe)	*Escherichia coli*
HCO70	NODE_20415_length_1500_cov_0.317553	cutE/lnt	BAC0115	Copper (Cu)	*Escherichia coli*
HCO11	NODE_206_length_28582_cov_4.454613	corB	BAC0643	Cobalt (Co), Magnesium (Mg)	*Escherichia coli*
HCO11	NODE_218_length_27224_cov_4.979592	cueO	BAC0103	Copper (Cu)	*Escherichia coli*
HCO11	NODE_21_length_122872_cov_3.596244	yodD	BAC0451	Cadmium (Cd)	*Escherichia coli*
HCO11	NODE_21_length_122872_cov_3.596244	cutC	BAC0114	Copper (Cu)	*Escherichia coli*
HCO11	NODE_21_length_122872_cov_3.596244	zinT/yodA	BAC0457	Cadmium (Cd), Zinc (Zn)	*Escherichia coli*
HCO72	NODE_22181_length_947_cov_0.339024	arsC	BAC0584	Arsenic (As), Antimony (Sb)	*Escherichia coli*
HCO64	NODE_22577_length_1304_cov_0.363636	cutA	BAC0113	Copper (Cu)	*Escherichia coli*
HCO11	NODE_234_length_26316_cov_5.732598	zntR/yhdM	BAC0462	Zinc (Zn)	*Escherichia coli*
HCO11	NODE_23_length_120452_cov_5.146603	pstA	BAC0315	Arsenic (As)	*Escherichia coli*
HCO11	NODE_23_length_120452_cov_5.146603	pstB	BAC0316	Arsenic (As)	*Escherichia coli*
HCO11	NODE_23_length_120452_cov_5.146603	pstS	BAC0318	Arsenic (As)	*Escherichia coli*
HCO11	NODE_23_length_120452_cov_5.146603	yieF	BAC0541	Chromium (Cr), Vanadium (V), Molybdenum (Mo)	*Escherichia coli*
HCO11	NODE_23_length_120452_cov_5.146603	pstC	BAC0317	Arsenic (As)	*Escherichia coli*
HCO64	NODE_24257_length_1244_cov_0.434199	corD	BAC0644	Cobalt (Co), Magnesium (Mg)	*Enterobacteriaceae*
HCO64	NODE_24353_length_1241_cov_0.394973	nikD	BAC0273	Nickel (Ni)	*Enterobacteriaceae*
HCO64	NODE_24494_length_1237_cov_0.301802	corA	BAC0086	Magnesium (Mg), Cobalt (Co), Nickel (Ni), Manganese (Mn)	*Enterobacteriaceae*
HCO64	NODE_24575_length_1234_cov_0.419151	arsR	BAC0594	Arsenic (As)	*Escherichia coli*
HCO11	NODE_24_length_118676_cov_4.756556	robA	BAC0334	Silver (Ag), Mercury (Hg), Cadmium (Cd)	*Escherichia coli*
HCO70	NODE_25004_length_1306_cov_0.711620	silF	BAC0345	Silver (Ag)	*Enterobacteriaceae*
HCO72	NODE_25629_length_868_cov_0.657220	cutA	BAC0113	Copper (Cu)	*Enterobacteriaceae*
HCO11	NODE_25_length_118301_cov_4.153139	modC	BAC0611	Tungsten (W), Molybdenum (Mo)	*Escherichia coli*
HCO11	NODE_25_length_118301_cov_4.153139	modE	BAC0608	Tungsten (W), Molybdenum (Mo)	*Escherichia coli*
HCO11	NODE_25_length_118301_cov_4.153139	modB	BAC0610	Tungsten (W), Molybdenum (Mo)	*Escherichia coli*
HCO11	NODE_25_length_118301_cov_4.153139	modA	BAC0609	Tungsten (W), Molybdenum (Mo)	*Escherichia coli*
HCO11	NODE_25_length_118301_cov_4.153139	zitB/ybgR	BAC0459	Zinc (Zn)	*Escherichia coli*
HCO64	NODE_26679_length_1169_cov_0.425144	mntR	BAC0253	Manganese (Mn), Magnesium (Mg)	*Escherichia albertii*
HCO64	NODE_27940_length_1134_cov_0.513406	zur/yjbK	BAC0470	Zinc (Zn)	*Enterobacteriaceae*
HCO70	NODE_28053_length_1211_cov_0.454797	rcnR/yohL	BAC0332	Cobalt (Co), Nickel (Ni), Iron (Fe)	*Escherichia coli*
HCO11	NODE_28_length_109031_cov_3.970185	nfsA	BAC0540	Chromium (Cr)	*Escherichia coli*
HCO11	NODE_29262_length_884_cov_0.225892	cadD	BAC0057	Cadmium (Cd), Zinc (Zn)	*Aggregatibacter sp. 2125159857*
HCO64	NODE_29337_length_1100_cov_0.445015	mntH/yfeP	BAC0251	Manganese (Mn), Iron (Fe), Cadmium (Cd), Cobalt (Co), Zinc (Zn)	*Enterobacteriaceae*
HCO11	NODE_2_length_338587_cov_4.047010	baeS	BAC0040	Zinc (Zn), Tungsten (W)	*Escherichia coli*
HCO11	NODE_2_length_338587_cov_4.047010	rcnR/yohL	BAC0332	Cobalt (Co), Nickel (Ni), Iron (Fe)	*Escherichia coli*
HCO11	NODE_2_length_338587_cov_4.047010	rcnB/yohN	BAC0331	Nickel (Ni), Cobalt (Co)	*Escherichia coli*
HCO11	NODE_2_length_338587_cov_4.047010	baeR	BAC0039	Zinc (Zn), Tungsten (W)	*Escherichia coli*
HCO11	NODE_2_length_338587_cov_4.047010	rcnA/yohM	BAC0330	Cobalt (Co), Nickel (Ni), Iron (Fe)	*Escherichia coli*
HCO70	NODE_3000_length_5153_cov_0.659968	arsC	BAC0584	Arsenic (As), Antimony (Sb)	*Escherichia coli*
HCO70	NODE_30662_length_1139_cov_0.663043	zinT/yodA	BAC0457	Cadmium (Cd), Zinc (Zn)	*Escherichia coli*
HCO11	NODE_30_length_106529_cov_4.863753	corD	BAC0644	Cobalt (Co), Magnesium (Mg)	*Escherichia coli*
HCO70	NODE_31421_length_1122_cov_0.271357	pcoB	BAC0304	Copper (Cu)	*Enterobacterales*
HCO72	NODE_32942_length_745_cov_0.579288	ygiW	BAC0445	Cadmium (Cd)	*Escherichia coli*
HCO72	NODE_3382_length_2860_cov_0.365532	corA	BAC0086	Magnesium (Mg), Cobalt (Co), Nickel (Ni), Manganese (Mn)	*Enterobacteriaceae*
HCO70	NODE_34074_length_1061_cov_0.327623	nikC	BAC0272	Nickel (Ni)	*Escherichia*

Sample	Contig ID	gene ID	BacMet ID	Compound	Contig taxonomic classification
HCO70	NODE_35872_length_1024_cov_0.329989	sodA	BAC0368	Selenium (Se)	*Enterobacteriaceae*
HCO11	NODE_3_length_324163_cov_3.938686	dsbB	BAC0137	Cadmium (Cd), Mercury (Hg)	*Escherichia coli*
HCO11	NODE_3_length_324163_cov_3.938686	bhsA/ycfR/comC	BAC0049	Copper (Cu)	*Escherichia coli*
HCO11	NODE_3_length_324163_cov_3.938686	ychH	BAC0434	Cadmium (Cd)	*Escherichia coli*
HCO11	NODE_3_length_324163_cov_3.938686	comR/ycfQ	BAC0076	Copper (Cu)	*Escherichia coli*
HCO11	NODE_40_length_88364_cov_5.283498	sodA	BAC0368	Selenium (Se)	*Escherichia coli*
HCO11	NODE_40_length_88364_cov_5.283498	dsbA	BAC0136	Cadmium (Cd), Zinc (Zn), Mercury (Hg)	*Escherichia coli*
HCO11	NODE_40_length_88364_cov_5.283498	glpF	BAC0181	Antimony (Sb), Arsenic (As)	*Escherichia coli*
HCO11	NODE_40_length_88364_cov_5.283498	fieF/yiip	BAC0167	Iron (Fe), Zinc (Zn), Cobalt (Co), Cadmium (Cd), Nickel (Ni)	*Escherichia coli*
HCO70	NODE_42277_length_917_cov_0.597468	modA	BAC0609	Tungsten (W), Molybdenum (Mo)	*Enterobacteriaceae*
HCO70	NODE_4296_length_4078_cov_0.667932	fieF/yiip	BAC0167	Iron (Fe), Zinc (Zn), Cobalt (Co), Cadmium (Cd), Nickel (Ni)	*Escherichia coli*
HCO11	NODE_42_length_87057_cov_3.574313	tehA	BAC0384	Tellurium (Te)	*Escherichia coli*
HCO11	NODE_42_length_87057_cov_3.574313	tehB	BAC0385	Tellurium (Te)	*Escherichia coli*
HCO72	NODE_4551_length_2416_cov_0.390127	modC	BAC0611	Tungsten (W), Molybdenum (Mo)	*Escherichia coli*
HCO72	NODE_4551_length_2416_cov_0.390127	modB	BAC0610	Tungsten (W), Molybdenum (Mo)	*Escherichia coli*
HCO70	NODE_46854_length_857_cov_0.497260	comR/ycfQ	BAC0076	Copper (Cu)	*Enterobacteriaceae*
HCO11	NODE_47_length_82245_cov_5.535242	corA	BAC0086	Magnesium (Mg), Cobalt (Co), Nickel (Ni), Manganese (Mn)	*Escherichia coli*
HCO11	NODE_4_length_311344_cov_4.443671	cueR/ybbI	BAC0105	Copper (Cu)	*Escherichia coli*
HCO11	NODE_4_length_311344_cov_4.443671	fetA/ybbL	BAC0165	Iron (Fe)	*Escherichia coli*
HCO11	NODE_4_length_311344_cov_4.443671	fetB/ybbM	BAC0166	Iron (Fe)	*Escherichia coli*
HCO11	NODE_4_length_311344_cov_4.443671	copA	BAC0725	Copper (Cu)	*Escherichia coli*
HCO70	NODE_5126_length_3643_cov_0.536974	zntR/yhdM	BAC0462	Zinc (Zn)	*Escherichia coli*
HCO64	NODE_52553_length_744_cov_0.372771	cueR/ybbI	BAC0105	Copper (Cu)	*Enterobacteriaceae*
HCO70	NODE_52574_length_794_cov_0.649175	dsbB	BAC0137	Cadmium (Cd), Mercury (Hg)	*Enterobacteriaceae*
HCO11	NODE_52_length_76755_cov_4.838701	cutF/nlpE	BAC0116	Copper (Cu)	*Escherichia coli*
HCO70	NODE_55118_length_771_cov_0.395963	terZ	BAC0392	Tellurium (Te)	*Escherichia coli*
HCO11	NODE_552_length_13688_cov_3.835853	fecD	BAC0163	Nickel (Ni), Cobalt (Co)	*Escherichia coli*
HCO11	NODE_552_length_13688_cov_3.835853	fecE	BAC0164	Nickel (Ni), Cobalt (Co)	*Escherichia coli*
HCO64	NODE_57055_length_701_cov_0.412892	yhcN	BAC0446	Cadmium (Cd)	*Escherichia coli*
HCO70	NODE_5754_length_3388_cov_0.583870	mgtA	BAC0087	Cobalt (Co), Magnesium (Mg)	*Escherichia coli*
HCO70	NODE_5904_length_3326_cov_0.780869	pstB	BAC0316	Arsenic (As)	*Escherichia coli*
HCO70	NODE_6322_length_3184_cov_0.420347	yodD	BAC0451	Cadmium (Cd)	*Escherichia coli*
HCO11	NODE_638_length_12393_cov_4.730556	silE	BAC0344	Silver (Ag)	*Enterobacter hormaechei*
HCO11	NODE_638_length_12393_cov_4.730556	silR	BAC0347	Silver (Ag)	*Enterobacter hormaechei*
HCO11	NODE_638_length_12393_cov_4.730556	silS	BAC0348	Silver (Ag)	*Enterobacter hormaechei*
HCO72	NODE_66455_length_460_cov_0.222222	cusF/cusX	BAC0110	Copper (Cu), Silver (Ag)	*Escherichia coli*
HCO72	NODE_6773_length_1926_cov_0.423569	arsR	BAC0594	Arsenic (As)	*Escherichia coli*
HCO70	NODE_6942_length_2998_cov_0.504006	fetA/ybbL	BAC0165	Iron (Fe)	*Escherichia coli*
HCO70	NODE_6942_length_2998_cov_0.504006	fetB/ybbM	BAC0166	Iron (Fe)	*Escherichia coli*
HCO11	NODE_6_length_283392_cov_5.057610	soxS	BAC0371	Zinc (Zn)	*Escherichia coli*
HCO11	NODE_6_length_283392_cov_5.057610	mgtA	BAC0087	Cobalt (Co), Magnesium (Mg)	*Escherichia coli*
HCO11	NODE_6_length_283392_cov_5.057610	zur/yjbK	BAC0470	Zinc (Zn)	*Escherichia coli*
HCO11	NODE_6_length_283392_cov_5.057610	cutA	BAC0113	Copper (Cu)	*Escherichia coli*
HCO70	NODE_7289_length_2909_cov_0.470525	zitB/ybgR	BAC0459	Zinc (Zn)	*Escherichia coli*
HCO70	NODE_74215_length_626_cov_0.428858	cutA	BAC0113	Copper (Cu)	*Escherichia coli*
HCO11	NODE_76_length_61052_cov_5.049602	pitA	BAC0312	Zinc (Zn), Tellurium (Te)	*Escherichia coli*
HCO70	NODE_7978_length_2750_cov_0.503241	corC	BAC0088	Cobalt (Co), Magnesium (Mg)	*Escherichia coli*

Sample	Contig ID	gene ID	BacMet ID	Compound	Contig taxonomic classification
HCO64	NODE_8062_length_2513_cov_0.492456	nikR	BAC0275	Nickel (Ni)	*Escherichia coli*
HCO64	NODE_8062_length_2513_cov_0.492456	nikE	BAC0274	Nickel (Ni)	*Escherichia coli*
HCO70	NODE_8201_length_2697_cov_0.501167	zur/yjbK	BAC0470	Zinc (Zn)	*Escherichia coli*
HCO70	NODE_8329_length_2672_cov_0.448723	pstA	BAC0315	Arsenic (As)	*Enterobacteriaceae*
HCO70	NODE_8329_length_2672_cov_0.448723	pstC	BAC0317	Arsenic (As)	*Enterobacteriaceae*
HCO70	NODE_8497_length_2637_cov_0.580080	soxS	BAC0371	Zinc (Zn)	*Escherichia coli*
HCO11	NODE_8_length_281109_cov_4.577293	dsbC	BAC0138	Copper (Cu)	*Escherichia coli*
HCO64	NODE_9105_length_2332_cov_0.280726	nikB	BAC0271	Nickel (Ni)	*Escherichia*
HCO70	NODE_91820_length_524_cov_0.191436	bhsA/ycfR/comC	BAC0049	Copper (Cu)	*Escherichia coli*
HCO70	NODE_9450_length_2468_cov_0.845365	glpF	BAC0181	Antimony (Sb), Arsenic (As)	*Escherichia coli*
HCO11	NODE_94_length_54277_cov_4.339612	mntR	BAC0253	Manganese (Mn), Magnesium (Mg)	*Escherichia coli*
HCO64	NODE_9919_length_2201_cov_0.501929	zraS/hydG	BAC0468	Zinc (Zn), Lead (Pb)	*Escherichia coli*

TABLE III

Kilobase per million sequenced reads (RPKM) abundances of the metal resistance genes identified in the Tunapuco and Yanomami

Group	Tunapuco				Yanomami											
ID	HCO11	HCO64	HCO70	HCO72	AH08	AH19	AL18	AL19	CA02	CA09	CA103	CA46	CA60	TA08	TA24	TA91
abeS	0	0	0	0	0	0	0	0	0	0	0	0	0	0.677	0	0
acrA	28.869	0	1.972	0	0	0	0	0	0	0	0	28.101	0	4.186	0	0
acrB	26.688	0	0	0	0	0	0	0	0	0	0	24.240	0	0	0	0
acrD/yffA	32.145	0	0	0	0	0	0	0	0	0	0	16.233	0	0	0	0
acrE/envC	30.372	2.349	0	0	0	0	9.370	0	0	0	0	17.394	0	6.736	0	0
acrF/envD	29.721	0	0	0	0	0	0	0	0	0	0	18.245	0	0	0	0
acrR/ybaH	30.926	0	2.189	4.981	0	0	0	0	0	0	0	21.582	0	2.378	0	0
actP/yjcG	30.485	0	0	0	0	0	0	0	0	0	0	18.992	0	0	0	0
arsB	27.575	0	0	0	0	0	0	0	0	0	0	17.227	0	9.656	0	0
arsC	31.519	0	3.505	4.344	0	0	8.934	0	0	0	0	14.905	0	13.130	5.105	0
arsR	32.551	2.548	2.742	2.815	0	0	0	0	0	0	0	23.193	0	6.088	0	0
asr	29.524	0	2.000	0	0	0	0	0	0	0	0	15.802	0	14.509	0	0
baeR	32.985	0	2.044	0	0	0	0	0	0	0	0	15.217	0	4.684	0	0
baeS	29.546	0	0	0	0	0	0	0	0	0	0	12.456	0	0	0	0
bcr	30.287	0	0	0	0	0	0	0	0	0	0	17.487	0	8.186	0	0
bhsA/ycfR/comC	35.731	0	1.296	4.965	0	0	0	0	0	0	0	20.579	0	6.842	0	0
cadD	1.758	0	0	0	0	0	0	0	0	0	0	0	0	0	0	0
cadX	0	0	0	0	0	0	0	0	0	0	0	0	0	0	5.095	0
chrA1	0	0	0	0	5.487	0	0	10.330	0	0	0	0	0	0	13.121	5.871
chrC	0	0	0	0	5.144	0	0	3.338	0	0	0	0	0	0	13.746	3.938
chrF	0	0	0	0	0	0	0	0	0	0	0	0	20.634	0	0	7.794
chtR	0	0	0	0	0	0	0	0	0	0	0	0	0	7.374	0	0
chtS	0	0	0	0	0	0	0	0	0	0	0	0	0	7.552	0	0
cnrA	0	0	0	0	4.483	0	0	4.111	0	0	0	0	0	0	14.406	6.057
cnrR/cnrX	0	0	0	0	5.275	0	0	3.963	0	0	0	0	0	0	13.510	8.945
comR/ycfQ	32.132	0	2.262	0	0	0	0	0	0	0	0	13.665	0	3.825	0	0
copA	28.480	0	0	0	0	0	0	0	0	0	0	14.990	0	0	0	0
copB	0	0	0	0	0	0	0	0	0	0	0	0	0	7.202	0	0
copC	0	0	0	0	0	0	0	1.890	0	0	0	0	0	0	0	0
copR	0	0	0	0	32.788	0	0	6.800	0	0	0	0	0	0	34.265	32.576
copY/tcrY	0	0	0	0	0	0	0	0	0	0	0	0	0	4.569	0	0
copZ	0	0	0	0	0	0	0	0	0	0	0	0	0	10.508	0	0

Group	Tunapuco				Yanomami											
ID	HCO11	HCO64	HCO70	HCO72	AH08	AH19	AL18	AL19	CA02	CA09	CA103	CA46	CA60	TA08	TA24	TA91
corA	42.778	2.055	0	2.535	0	1.982	0	0	0	0	0	21.814	0	9.713	0	0
corB	27.688	0	0	0	0	0	0	0	0	0	0	17.184	0	3.436	0	0
corC	32.032	0	2.803	0	0	0	0	0	0	0	0	18.588	0	6.597	0	0
corD	40.292	2.187	0	0	0	0	0	0	0	0	0	23.675	0	4.344	0	0
cpxA	36.682	0	2.613	4.147	0	0	0	0	0	0	0	18.345	0	6.179	0	0
cpxR	36.841	0	0	0	0	0	0	0	0	0	0	23.218	0	5.726	0	0
cueO	30.041	0	0	0	0	0	0	0	0	0	0	13.905	0	5.889	0	0
cueR/ybbI	39.295	1.658	0	0	0	0	0	0	0	0	0	13.952	0	3.845	0	0
cusA/ybdE	27.696	0	0	0	0	0	0	0	0	0	0	13.057	0	3.770	0	0
cusB	32.172	0	0	0	0	0	0	0	0	0	0	11.850	0	1.677	0	0
cusC/ylcB	32.966	0	0	0	0	0	0	0	0	0	0	11.557	0	0	0	0
cusF/cusX	34.604	1.488	0	3.420	0	0	0	0	0	0	0	13.643	0	4.007	0	0
cusR/ylcA	28.713	0	2.729	0	0	0	0	0	0	0	0	12.244	0	6.096	0	0
cusS	26.705	0	0	0	0	0	0	0	0	0	0	15.645	0	0	0	0
cutA	41.381	1.552	1.464	5.039	0	3.178	0	0	0	0	0	0	0	4.541	0	0
cutC	25.620	0	0	0	0	0	0	0	0	0	0	20.920	0	3.023	0	0
cutE/lnt	29.689	0	1.932	0	0	0	0	0	0	0	0	18.370	0	5.868	0	0
cutF/nlpE	37.205	0	0	0	0	0	0	0	0	0	0	18.438	0	0	0	0
czcC	0	0	0	0	0	0	0	0	0	0	0	0	0	0	3.766	0
dsbA	34.359	0	0	0	0	1.916	0	0	0	3.556	0	15.583	0	15.387	0	0
dsbB	22.078	0	2.329	0	0	0	37050.779	0	0	0	0	22.214	0	14.883	0	0
dsbC	32.132	1.508	0	0	0	0	0	0	0	0	0	18.746	0	4.258	0	0
emmdR	18.100	0	1.956	0	0	2.777	0	0	0	0	0	10.490	0	21.183	0	0
emrA	35.792	0	2.800	0	0	0	0	0	0	0	0	17.359	0	3.194	0	0
emrB	28.127	0	0	0	0	0	0	0	0	0	0	19.063	0	4.386	0	0
emrD	40.080	0	0	0	0	0	0	0	0	0	0	17.368	0	6.366	0	0
emrE/mvrC	25.577	0	1.464	0	0	0	8.331	0	0	0	0	20.793	0	6.668	0	0
emrK	0	0	2.554	0	0	0	0	0	0	0	0	21.278	0	0	0	0
emrR	33.589	0	3.111	0	0	0	3.987	0	0	0	0	16.596	0	2.996	0	0
emrY	0	0	0	0	0	2.536	24.990	0	0	0	0	12.359	0	0	0	0
evgA	0	0	3.520	0	0	0	0	0	0	0	0	14.774	0	27.893	0	0
evgS	0	0	0	0	0	0	0	0	0	0	0	16.486	0	13.878	0	0
fabI	24.384	0	1.419	2.791	0	0	0	0	0	0	0	16.476	0	0	0	0
fecD	24.605	0	0	0	0	0	6.234	0	0	0	0	0	0	0	0	0
fecE	27.138	0	0	0	0	0	0	0	0	0	0	0	0	0	0	0
fetA/ybbL	31.479	0	3.634	2.157	0	0	0	0	0	0	0	15.944	0	6.585	0	0
fetB/ybbM	29.033	0	3.254	0	0	0	0	0	0	0	0	15.228	0	7.039	0	0
fieF/yiip	35.509	0	2.976	0	0	0	0	0	0	0	0	21.428	0	5.853	0	0
gadA	0	2.398	0	0	0	2.499	0	0	0	0	0	0	0	12.387	0	0
gadC/xasA	17.874	0	0	0	0	0	0	0	0	0	0	19.849	0	3.875	0	0
gadE/yhiE	31.912	0	2.464	0	0	0	7.901	0	0	0	0	20.097	0	14.054	0	0
gadW/yhiW	27.215	2.061	3.584	3.905	0	0	0	0	0	0	0	19.709	0	7.532	0	0
gadX	28.178	2.460	0	0	0	0	27.430	0	0	0	0	24.714	0	9.019	0	0
galE	0	0	0	0	0	0	11.739	0	0	0	0	0	0	0	6.267	0
glpF	36.835	0	3.468	0	0	0	0	0	0	0	0	15.366	0	6.976	0	0
hdeA/yhiB	27.081	1.128	0	3.420	0	1.887	0	0	0	0	0	23.752	0	6.318	0	0
hdeB/yhiC	25.715	2.439	2.202	2.939	0	2.118	3.181	0	0	0	0	29.871	0	11.504	0	0
ibpA	34.853	0	4.328	4.126	0	0	3.230	0	0	0	0	20.823	0	11.775	0	0
ibpB	37.371	2.278	5.221	4.977	0	0	0	0	0	0	0	15.885	0	15.789	0	0
iclR	30.501	0	3.716	0	0	0	0	0	0	0	0	20.114	0	8.256	0	0
kpnE	0	0	0	0	0	0	0	0	0	0	0	0	0	0	2.094	0
kpnO	0	0	0	0	0	0	0	0	0	0	0	38.399	0	0	0	0

Group	Tunapuco				Yanomami											
ID	HCO11	HCO64	HCO70	HCO72	AH08	AH19	AL18	AL19	CA02	CA09	CA103	CA46	CA60	TA08	TA24	TA91
marA	17.985	0	1.965	0	0	1.460	3.458	0	0	5.654	0	21.122	0	28.331	0	0
marR	14.512	0	2.244	0	0	2.270	0	0	0	0	0	21.014	0	26.543	0	0
mdfA/cmr	23.730	0	0	0	0	0	0	0	0	0	0	16.603	0	0	0	0
mdtA/yegM	27.058	0	0	0	0	0	0	0	0	0	0	15.394	0	0	0	0
mdtB/yegN	26.727	0	0	0	0	0	0	0	0	0	0	17.343	0	0	0	0
mdtC/yegO	26.499	0	0	0	0	0	0	0	0	0	0	13.302	0	0	0	0
mdtE/yhiU	29.333	2.022	3.030	0	0	0	0	0	0	0	0	16.024	0	6.248	0	0
mdtF/yhiV	25.871	0	0	0	0	0	0	0	0	0	0	23.176	0	0	0	0
mdtG/yceE	21.233	0	0	0	0	0	0	0	0	0	0	17.353	0	4.391	0	0
mdtI/ydgE	31.579	0	0	0	0	0	0	0	0	0	0	13.103	0	11.196	0	0
mdtJ/ebrB/ydgF	32.305	0	2.856	0	0	0	0	0	0	0	8.429	14.133	0	11.918	0	0
mdtK/ydhE	20.711	0	2.336	0	0	0	0	0	0	0	0	19.500	0	11.540	0	0
mdtM/yjiO	29.825	0	3.251	0	0	0	0	0	0	0	0	19.399	0	5.656	0	0
mdtN/yjcR	34.954	0	0	4.276	0	0	0	0	0	0	0	15.540	0	0	0	0
merA	0	0	0	0	4.672	0	0	0	0	0	0	0	0	0	18.287	4.346
merD	0	0	0	0	6.041	0	0	2.553	0	0	0	0	0	0	24.293	9.334
merE	0	0	0	0	4.665	0	0	3.504	0	0	0	0	0	0	25.911	7.103
merF	0	0	0	0	0	0	0	0	0	0	0	0	0	0	3.900	1.000
merP	0	0	0	0	2.170	0	1.863	2.445	0	0	0	0	0	0	5.562	2.153
merR	0	0	0	0	3.449	0	0	0	0	0	0	0	0	0	8.676	5.384
merR2	0	0	0	0	3.706	0	0	2.983	0	0	0	0	0	0	10.735	0
merT	0	0	0	0	3.937	0	0	1.331	0	0	0	0	0	0	5.103	3.244
mgtA	30.614	0	2.814	0	0	0	0	0	0	0	0	16.662	0	7.765	0	0
mntH/yfeP	26.688	2.244	0	0	0	0	0	0	0	0	0	14.645	0	8.822	0	0
mntP/yebN	0	0	0	0	0	0	0	0	0	0	0	17.672	0	0	0	0
mntR	34.899	1.927	0	4.313	0	0	4.590	0	0	0	0	8.947	0	9.539	0	0
modA	29.517	0	3.636	0	0	0	0	0	0	0	0	15.982	0	6.298	0	0
modB	28.753	0	0	3.919	0	0	0	0	0	0	0	15.271	0	5.652	21291.383	0
modC	28.669	0	0	2.554	0	0	0	0	0	0	0	13.904	0	4.652	0	0
modE	27.812	0	0	0	0	0	7.561	0	0	0	0	18.939	0	0	0	0
nfsA	23.976	0	0	0	0	0	0	0	0	0	0	13.211	0	5.891	0	0
nikA	31.873	0	2.607	0	0	0	0	0	0	0	0	13.988	0	4.952	0	0
nikB	31.916	2.386	0	4.820	0	0	0	0	0	0	0	14.104	0	3.204	0	0
nikC	34.602	0	2.567	0	0	0	0	0	0	0	0	21.592	0	3.222	0	0
nikD	32.614	2.161	0	4.279	0	0	0	0	0	0	0	20.034	0	0	0	0
nikE	33.400	2.456	0	0	0	0	0	0	0	0	0	20.958	0	0	0	0
nikR	42.872	2.991	0	0	0	0	0	0	0	0	0	20.424	0	2.808	0	0
opmD/nmpC	30.440	0	0	0	0	0	0	0	0	0	0	0	0	0	0	0
ostA/lptD	33.145	0	0	0	0	0	0	0	0	0	0	23.452	0	3.129	0	0
oxyRkp	0	2.394	0	0	0	0	0	0	0	0	0	0	0	7.567	0	0
pcoA	25.464	0	0	0	0	0	0	0	0	0	0	0	0	0	0	0
pcoB	24.911	0	1.791	0	0	0	0	0	0	0	0	0	0	0	0	0
pcoC	30.770	0	0	0	0	0	0	0	0	0	0	0	0	0	0	0
pcoD	32.431	0	0	0	0	0	0	0	0	0	0	0	0	0	0	0
pcoE	24.878	0	0	0	0	0	0	0	0	0	0	0	0	0	0	0
pcoR	28.251	0	0	0	0	0	0	0	0	0	0	0	0	0	0	0
pcoS	28.680	0	0	0	0	0	0	0	0	0	0	0	0	0	0	0
pdrM	0	0	0	0	0	0	0	0	0	0	0	0	0	0	6.997	0
phoB	25.647	0	0	0	0	0	0	0	0	0	0	14.987	0	3.589	0	0
phoR	21.673	0	0	0	0	0	0	0	0	0	0	16.616	0	4.952	0	0
pitA	35.204	0	0	0	0	0	0	0	0	0	0	18.628	0	9.442	0	0

Group	Tunapuco				Yanomami											
ID	HCO11	HCO64	HCO70	HCO72	AH08	AH19	AL18	AL19	CA02	CA09	CA103	CA46	CA60	TA08	TA24	TA91
pstA	33.850	0	3.016	0	0	0	0	0	0	0	0	16.064	0	3.628	0	0
pstB	37.025	0	5.305	0	7.433	0	0	3.104	0	0	0	32.955	0	10.343	21.648	8.165
pstC	31.835	0	2.489	0	0	0	0	0	0	0	0	17.761	0	0	0	0
pstS	33.689	0	0	0	0	0	0	0	0	0	0	17.535	0	3.796	0	0
qacC/qacD/smr	0	0	0	0	38.102	0	0	0	0	0	0	0	0	0	0	25.520
rcnA/yohM	24.898	0	0	0	0	0	0	0	0	0	0	19.771	0	11.877	7.940	0
rcnB/yohN	31.084	1.819	2.302	0	0	2.161	17.639	5.176	0	0	0	20.038	0	22.642	10.600	0
rcnR/yohL	27.161	0	3.282	0	0	0	0	0.876	0	0	0	14.135	0	16.165	0	0
robA	34.898	0	0	0	0	0	0	0	0	0	0	26.141	0	2.595	0	0
rpoS	35.721	0	0	0	0	0	0	0	0	0	0	19.237	0	7.132	0	0
silA	27.828	0	0	0	0	0	0	0	0	0	0	0	0	0	0	0
silB	28.673	0	0	0	0	0	0	0	0	0	0	0	0	0	0	0
silC	30.558	0	0	0	0	0	0	0	0	0	0	0	0	0	0	0
silE	35.627	0	0	0	0	0	0	0	0	0	0	0	0	0	0	0
silF	30.570	0	3.796	0	0	0	0	0	0	0	0	0	0	0	0	0
silP	28.734	0	0	0	0	0	0	0	0	0	0	0	0	0	0	0
silR	23.542	0	0	0	0	0	0	0	0	0	0	0	0	0	0	0
silS	25.123	0	0	0	0	0	0	0	0	0	0	0	0	0	0	0
sitC	0	0	0	0	0	0	0	0	0	0	0	0	0	7.596	0	0
sodA	45.018	0	2.525	0	0	0	0	0	0	0	0	17.540	0	6.445	0	0
sodB	34.089	0	0	0	0	0	0	0	0	0	0	23.841	0	6.260	0	0
soxR	39.218	0	3.532	4.168	0	1.951	0	0	0	0	0	14.832	0	10.153	0	0
soxS	35.874	1.392	3.226	0	0	0.809	0	0	0	0	0	15.373	0	7.444	0	0
sugE	0	0	4.115	4.038	0	0	0	3.327	0	0	0	0	0	0	0	0
tehA	20.539	0	1.783	0	0	0	0	0	0	0	0	20.397	0	6.842	0	0
tehB	26.822	0	0	0	0	0	8.468	0	0	0	0	17.416	0	10.712	0	0
terZ	0	0	1.685	0	0	0	0	0	0	0	0	0	0	0	0	0
tolC	25.993	0	3.061	0	0	0	0	0	0	0	0	21.997	0	0	0	0
ychH	29.091	0	2.765	0	0	0	4.793	1.116	0	0	0	33.155	0	6.070	0	0
yddg/emrE	23.289	0	0	0	0	0	0	0	0	0	0	18.587	0	3.724	0	0
ydeI	19.887	0	2.320	0	0	4.340	22.247	2.774	0	0	0	11.908	0	17.628	0	0
ydeO	19.856	0	2.841	0	0	0	60.475	6.737	0	0	0	17.994	0	13.199	0	0
ydeP	16.656	0	0	0	0	0	0	0	0	0	0	22.579	0	9.385	0	0
yfeB	0	0	0	0	0	0	12.655	0	0	0	0	0	0	0	0	0
ygiW	36.460	0	3.229	3.037	0	0	0	0	0	0	0	13.788	0	6.137	0	0
yhcN	45.806	1.432	4.503	4.971	0	1.995	0	0	0	0	0	13.380	0	10.100	0	0
yieF	29.866	0	2.370	0	0	0	0	0	0	0	0	17.907	0	12.037	0	0
yjaA	0	0	0	0	0	0	0	0	0	0	0	0	0	2.830	0	0
ymgB/ariR	21.016	1.970	0	0	0	2.017	61.254	0	0	0	0	23.165	0	22.295	0	0
yodD	29.930	0	2.909	0	0	0	0	0	0	0	0	22.155	0	15.995	0	0
yqjH	33.400	0	2.635	0	0	0	9.681	0	0	0	0	14.382	0	4.293	0	0
zinT/yodA	20.726	0	2.765	0	0	0	18.645	0	0	0	0	20.526	0	7.094	0	0
zitB/ybgR	28.188	0	2.853	0	0	0	37.842	3.472	0	0	0	24.986	0	6.973	0	0
zntA/yhhO	35.815	0	0	0	0	0	0	0	0	0	0	16.142	0	4.611	0	0
zntR/yhdM	35.047	2.646	3.505	0	0	0	5.554	0	0	0	0	23.449	0	9.155	0	0
znuA/yebL	28.324	0	2.269	0	0	0	0	0	0	0	0	12.506	0	7.539	0	0
znuB/yebI	29.958	0	0	0	0	2.170	10.673	0	0	0	0	18.489	0	0	0	0
znuC/yebM	33.135	0	2.271	0	0	0	14.422	0	0	0	0	20.635	0	7.942	0	0
zraR/hydH	36.196	0	2.871	0	0	0	0	0	0	0	0	15.665	0	0	0	0
zraS/hydG	36.238	2.487	0	0	0	0	0	0	0	0	0	17.768	0	3.019	0	0
zupT/ygiE	23.950	0	0	0	0	0	0	0	0	0	0	11.704	0	9.746	0	0
zur/yjbK	38.449	1.748	1.755	2.483	0	0	0	2.414	8.575	0	0	23.019	0	13.426	1.870	0
